# Theoretically Universal, Practically Unequal: Socio‐Economic Inequalities in Healthcare Access for Long Covid‐19 Patients in Austria

**DOI:** 10.1111/hex.70553

**Published:** 2026-01-08

**Authors:** Peter Gamillscheg‐Müllner, Agata Łaszewska, Sophie Diexer, Kathryn Hoffmann, Judit Simon, Susanne Mayer

**Affiliations:** ^1^ Department of Health Economics, Center for Public Health Medical University of Vienna Vienna Austria; ^2^ Department of Primary Care Medicine, Center for Public Health Medical University of Vienna Vienna Austria; ^3^ Department of Psychiatry, Warneford Hospital University of Oxford Oxford UK

**Keywords:** barriers, demographic, facilitators, healthcare access, inequalities, long Covid‐19, socio‐economic, universal healthcare system, unmet healthcare needs

## Abstract

**Background:**

Long Covid‐19 (LC) patients have substantial treatment and care needs, yet research has shown that the majority of them experience healthcare access barriers. While qualitative studies indicate socio‐economic and demographic access inequalities among LC patients, quantitative evidence remains limited. This study aims to assess socio‐economic inequalities in healthcare access among LC patients in Austria, focusing on self‐perceived barriers, facilitators and unmet healthcare needs.

**Methods:**

Retrospective cross‐sectional data were collected from adult LC patients through online and paper‐based surveys (10‐12/2024), following a prior qualitative study. The survey assessed 47 barriers and 10 facilitators based on Levesque's ‘access to care’ framework, along with unmet healthcare needs overall and related to general practitioner (GP), specialist and hospital care. Overall barrier and facilitator scores were calculated. Inequalities related to gender, age, urbanicity, health‐related background through training/employment, complementary private health insurance, and economic situation were examined in linear, logistic and ordered logistic regressions, controlling for clinical and demographic variables.

**Results:**

Overall, 433 LC patients completed the survey. Participants living in urban areas, with complementary private health insurance, or in a good economic situation reported fewer barriers, reflected in statistically significantly lower overall barrier scores. Income‐related inequalities emerged particularly in relation to barriers in GP care, including not being taken seriously, attribution of symptoms to mental health conditions, burdensome costs, short consultation times, and limited availability of telemedicine or home visits. Facilitator scores, in contrast, did not differ by socio‐economic factors. Living in a rural area was associated with a higher probability of unmet healthcare needs related to GP and specialist care. A poor economic situation was associated with a higher probability of reporting unmet needs related to specialist and hospital care. No evidence of gender‐based inequalities was found.

**Conclusions:**

Our findings reveal enhanced inequalities in LC healthcare access in an otherwise universal healthcare system. Contrary to prior research, we find income‐related inequalities in GP access. Future policy efforts in Austria should consider that central case management through GP care may not be the most optimal set‐up, especially without improved information, training, support and specialist referral opportunities.

**Patient or Public Contribution:**

The design of the survey and the hypotheses on healthcare access barriers and facilitators were directly informed by qualitative interviews from previous work with long Covid‐19 (LC) patients, who shared their lived experiences with diagnosis, treatment and navigating the healthcare system. Additionally, LC patients piloted the survey before its launch and provided feedback. Representatives of patient LC groups and individual patients contributed to participant recruitment by sharing study materials within their networks.

**Abbreviations**
BACE: Barriers to Access to Care Evaluation scale
EU‐SILC: EU Statistics on Income and Living Conditions
GP: general practitioner
LC: long Covid‐19
NICE: National Institute for Health and Care Excellence
OECD: Organisation for Economic Co‐operation and Development
STROBE: Strengthening the Reporting of Observational Studies in Epidemiology
WHO: World Health Organization

## 1 Background

More than 2 years after the World Health Organization (WHO) declared the end of the Covid‐19 global health emergency in May 2023 [1], the corresponding chronic condition, long Covid‐19 (LC), still poses a substantial challenge to health and social systems globally. The precise prevalence of LC is difficult to determine; however, even conservative estimates arrive at a cumulative global incidence of 400 million in 2023 [2]. This figure is particularly pressing as recent research finds that more than two‐thirds of working‐age patients do not recover in the second year of their disease [3]. With public and private budgets under pressure due to slow economic recovery, the medical costs and productivity losses due to LC also present an urgent call to action for decision‐makers [4, 5].

LC is characterised by a wide range of possible physical and mental symptoms such as fatigue, neurocognitive dysfunctions and impaired activity and functioning [6]. Expectedly, previous research found that LC patients have an increased healthcare utilisation and corresponding medical costs compared to those without a LC diagnosis and to their pre‐Covid‐19 diagnosis period [7, 8, 9, 10]. International research points to not only the severity of the acute Covid‐19 illness being associated with increased LC healthcare utilisation, but also socio‐economic and demographic factors such as higher age, female gender and comorbidities [10, 11]. These associations are coherent with the risk factors to develop LC in the first place, as research shows similar associations and particularly increased LC risk to those most deprived [12, 13, 14].

While the increased healthcare utilisation by LC patients might imply that their increased needs are sufficiently catered for, the opposite seems to be the case. Prior research finds increased unmet healthcare needs in this patient population compared to patients who did not develop LC after Covid‐19 and those who never tested positive for Covid‐19 [15]. Moreover, exploratory qualitative studies point to substantial difficulties and access barriers encountered by LC patients in their efforts to obtain appropriate care. This issue persists across health systems, although the types of barriers described vary, partly depending on the existence or absence of gatekeepers in the form of general practitioners (GPs) [16, 17, 18, 19, 20]. Prior qualitative research also indicates prevailing socio‐economic and demographic differences in healthcare access barriers faced by LC patients [16]. However, to date, only one Dutch study has quantitatively investigated these differences, identifying factors such as age and education as affecting patients' experiences [21]. Considering the long‐standing policy priority to reduce inequalities and achieve equal, universal access to healthcare internationally, further research in this regard will help fill the existing evidence gap and support the development of targeted policy recommendations [22, 23].

The aim of this study is to examine socio‐economic and demographic inequalities in healthcare access barriers, access facilitators and unmet healthcare needs for LC in Austria. Drawing on a mixed‐methods research approach, we acknowledge the comparative novelty of the LC condition in a research context and build on the findings of a recently published qualitative study [16]. Specifically, we previously identified gender, age, urbanicity, health‐related background through training or employment, complementary private health insurance, and economic situation to potentially affect the healthcare access of LC patients [16]. In this context, Austria presents an interesting case, given its theoretically nearly universal healthcare system with 99.9% statutory health insurance coverage. At the same time, a growing private (non‐contracted) healthcare provider sector [24, 25] exists, with approximately 38% of the population having complementary private health insurance [25]. Generally, unmet healthcare needs are low in Austria, and there is no GP gatekeeping to steer access to specialist outpatient care [26, 27, 28]. Nevertheless, there is evidence of some existing general socio‐economic and demographic differences in healthcare utilisation and health outcomes [29, 30, 31].

## 2 Methods

### 2.1 Underlying Framework

**Figure 1:**
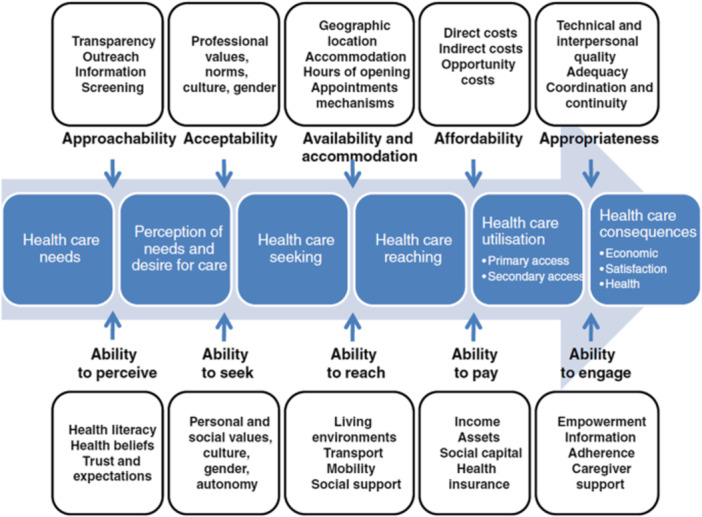
Access to healthcare framework by Levesque et al. [23]. Permission to use this conceptual visualisation was obtained from Jean‐Frederic Levesque.

The framework used in the qualitative first part of this research [16] and subsequently also applied in the survey developed for this study was established by Levesque et al. [32] and previously employed in other empirical quantitative research [33, 34]. The framework conceptualises ‘access to care’ as a stepwise progression spanning from healthcare needs to healthcare consequences across five dimensions at the interfaces of the involved process steps. The framework includes both a health system and population perspective, as each dimension is accompanied by a corresponding ability as visualised in Figure 1. Barriers and facilitators can occur at any of the dimensions, impeding or aiding advancement to the next step. For example, a patient might be able to perceive a need, seek care and reach it, but then end up not utilising any services as they would not be covered by the patient's health insurance. The direct costs in this case thus present a barrier in the dimension of affordability [32].

### 2.2 Survey Development and Data Collection

Using the ‘access to care’ framework [32], the previous qualitative study [16] identified potential barriers and facilitators to healthcare access for LC patients in Austria, leading to the development of a list of 47 barriers and 10 facilitators. Furthermore, the qualitative study [16] identified six socio‐economic and demographic characteristics that may affect the patient experience: gender, age, urbanicity, health‐related background, complementary private health insurance, and economic situation. Health‐related background was defined as having knowledge of the medical field through training or employment (e.g., nurses and veterinarians). Provided examples for this question included paramedics or emergency medical technicians, nurses or physicians and also related fields such as veterinarians or pharmacists. The content of the survey for this study was developed based on these findings. The unmet healthcare needs assessment was based on the EU Statistics on Income and Living Conditions (EU‐SILC) [35], while relevant research such as the Barriers to Access to Care Evaluation scale (BACE) informed the question design [36, 37, 38]. Experienced symptoms were assessed based on the questionnaire used in one Austrian region for initial LC assessment [39], which was refined based on expert feedback collected from Medical University of Vienna internal LC experts. The survey was successfully piloted with two LC patients.

The survey was conducted in German, both online via SoSci Survey and paper‐based. The study's scope was to recruit a nationwide Austrian sample of acute and recovered LC patients of sufficient size to enable robust statistical analysis. Survey details including the survey link were distributed via (i) the patient support groups *Long Covid Austria* and *Long COVID und ME/CFS Tirol*, (ii) 64 physicians (both GPs and specialists) recommended by LC patients on the *Long Covid Austria* website [40], and (iii) 59 primary care centres to also reach patients who might not be aware of the LC support groups. To ensure anonymity, responses could not be linked to distribution channels. At the time of the study, there were roughly 3400 members in the *Long Covid Austria* patient support group on Facebook. However, the overall achieved outreach of the distribution effort across the three channels cannot be quantified.

The study followed a convenience sampling approach, so every eligible person with access to the survey could participate. Eligibility criteria included being at least 18 years of age, having sufficient German language skills to participate in the survey, experiencing either acute or recently recovered LC (within 3 months prior to the survey), and providing informed consent to participate. The LC status was patient‐reported, and no formal diagnosis was required, as getting a formal diagnosis itself could prove a barrier [16]. In the explanatory text of the inclusion criteria, the LC definition by the UK's National Institute for Health and Care Excellence (NICE) was provided for guidance [41]. To reduce participation burden, the survey could be paused at any time. At the end of the survey, participants could opt to enter a lottery to win one of three 50€ vouchers. Data collection took place between October and December 2024.

### 2.3 Variables

The survey included sections on the participants' clinical characteristics and socio‐economic and demographic characteristics, followed by a list of healthcare access barriers, facilitators and unmet healthcare needs. The full survey is provided in Supporting Material 1. Outcome variables included perceptions of 47 barriers and 10 facilitators individually, overall barrier and facilitator scores, and unmet healthcare needs, both overall and separately for GP, specialist and hospital care. Survey participants were asked to rate each barrier and facilitator as either not applicable (e.g., if a hospital‐related barrier for someone who never visited a hospital for LC), no problem/not helpful, minor problem/somewhat helpful or major problem/very helpful. This question design was based on the validated BACE instrument [36]; however, we added the ‘not applicable’ option. Overall barrier and facilitator scores were calculated by scoring ‘not applicable’ and ‘no problem/not helpful’ as zero, ‘minor problem/somewhat helpful’ as one and ‘major problem/very helpful’ as two, applied to each of the 47 individual barriers/10 facilitators, respectively. A higher barrier score indicates a higher burden, while a higher facilitator score reflects higher support. For unmet healthcare needs, participants were first asked whether they had ever incurred an unmet healthcare need related to LC. In case they did, the following questions asked for unmet healthcare needs regarding GP, specialist and hospital care. In those questions, participants were also asked to choose the most applicable of eight reasons [35] or indicate no unmet healthcare needs. Included reasons for unmet healthcare needs were financial reasons, waiting times, no time due to personal or professional obligations, insufficient accessibility, fear, wanting to wait for improved condition, no knowledge of a good physician/hospital, or other [35].

Explanatory variables of interest were gender, age, urbanicity, health‐related background, perceived household economic situation during LC, and complementary private health insurance. Control variables included whether patients lived in a federal state offering central LC care co‐ordination, highest level of education, migration background, relationship status and clinical characteristics (disease onset, disease duration, number of perceived severe LC symptoms, and prior chronic physical and mental health conditions), which were found to be associated with either healthcare access [42, 43, 44, 45, 46] or LC risk [47, 48, 49, 50, 51] in prior research. Detailed definitions of all included variables are provided in Table 1.

**Table 1:** Variable description.

	Variable	Type	Range	Measurement
*Outcome variables*	Individual barriers	Ordinal	Not applicable, no problem, minor problem, major problem	Participants were asked to assess 47 barriers as to whether they were applicable to them and, if so, to what extent they did or did not pose a problem.
	Individual facilitators	Ordinal	Not applicable, not helpful, somewhat helpful, very helpful	Participants were asked to assess 10 facilitators as to whether they were applicable to them and, if so, to what extent they were or were not helpful.
	Overall barrier score	Continuous	0–94	Summary variable generated to assess the overall barrier burden, counting ‘not applicable’ and ‘no problem’ as zero, ‘minor problem’ as one, and ‘major problem’ as two for each of the 47 barriers.
	Overall facilitator score	Continuous	0–20	Summary variable generated to assess the overall facilitator score, counting ‘not applicable’ and ‘not helpful’ as zero, ‘somewhat helpful’ as one and ‘very helpful’ as two for each of the 10 facilitators.
	Overall unmet healthcare needs	Binary	No unmet healthcare needs, unmet healthcare needs	Participants were asked to indicate whether they could not access one or more medical services required due to LC at all.
	Unmet GP healthcare needs	Binary	No unmet GP healthcare needs, unmet GP healthcare needs	Binary variable generated based on whether participants indicated that they could not access one or more GP services required due to LC at all for any reason.
	Unmet specialist healthcare needs	Binary	No unmet specialist healthcare needs, unmet specialist healthcare needs	Binary variable generated based on whether participants indicated that they could not access one or more specialist services required due to LC at all for any reason.
	Unmet hospital healthcare needs	Binary	No unmet hospital healthcare needs, unmet hospital healthcare needs	Binary variable generated based on whether participants indicated that they could not access one or more hospital services required due to LC at all for any reason.
*Explanatory variables of interest*	Gender	Categorical	Female, Male, Diverse	
	Age	Continuous/ordinal	18–30, 31–40, 41–50, 51–60, > 60	Participants were asked to provide their age, which was recoded into an ordinal variable consisting of five age groups for analyses.
	Urbanicity	Binary	Rural, urban	Participants were asked to describe their residence as rural or urban.
	Health‐related background	Binary	No health‐related background, health‐related background	Participants were asked whether they had a health‐related background, with examples given such as training or employment in medical fields (e.g., nurses and doctors), but also related fields (e.g., veterinary sciences) or activities in medical fields without medical training (e.g., pharmaceutical industry).
	Complementary insurance	Binary	No complementary private health insurance, complementary private health insurance	Participants were asked whether they had complementary private health insurance.
	Household economic situation during LC	Ordinal	Very good/good, medium, bad/very bad	Participants were asked to give an indication about their household's economic situation during their disease, which was recoded to three levels, combining good and very good, and bad and very bad.
*Control variables*	Central co‐ordination	Binary	No central co‐ordination available, central co‐ordination available	Binary variable generated based on federal regions provided by participants, as two out of nine (Tyrol and Vorarlberg) offer central LC co‐ordination.
	Education	Ordinal	Compulsory school/vocational training/vocational school, A‐level equivalent, university degree/other degree higher than A‐levels	Participants were asked to provide their highest attained level of education, which was recoded to lower than A‐level equivalent, A‐level equivalent and higher than A‐level equivalent.
	Migration background	Binary	No migration background, migration background	Participants were asked whether they had a migration background.
	Relationship status	Categorical/binary	Single, in a relationship living together, in a relationship living separately	Participants were asked whether they were single, including widowed and divorced, in a relationship, including any form and living together or separately, which was recoded to binary (single/in a relationship).
	Chronic physical health condition(s)	Ordinal/binary	No chronic condition, one chronic condition, multiple chronic conditions	Participants were asked whether they had one or more chronic physical health conditions prior to contracting Covid‐19, defined as conditions lasting at least six months, such as diabetes, asthma or hypertension, which was recoded to binary (no/one or more chronic physical health conditions).
	Mental health condition(s)	Binary	No mental health condition, mental health condition	Participants were asked whether they were undergoing treatment or received support for a mental health condition prior to contracting Covid‐19, such as anxiety, depression or bipolar disorder.
	Number of severe LC symptoms	Continuous	0–11 severe symptoms	Summary variable generated as a proxy of overall symptom burden, counting all symptoms individually perceived as highest/one point below highest severity on a five‐point Likert scale. Overall, 11 symptoms were assessed based on the initial LC assessment questionnaire used in one Austrian federal state (Tyrol) [39] and expert feedback provided by experts (persistent fatigue, post‐exertional malaise, breathing difficulties, muscle/joint problems, digestive problems, cognitive impairment, circulatory problems/palpitations, sleep problems, odour and taste disorders, headaches, anxiety/depression/somatisation).
	Disease onset quarter	Continuous	Q1 2020 to Q4 2024	Variable generated based on free‐text input asking for LC onset.
	Disease duration	Continuous	1–60 months	Variable generated based on free‐text input asking for LC onset and if recovered duration, calculating duration in months, either as provided or until the date of survey participation.

*Note:* Participants could always opt not to answer questions, which were omitted in the variable ranges for improved readability.

### 2.4 Analysis

The study sample characteristics were described using frequencies and percentages. Linear, ordered logistic, and logistic regressions were employed to assess socio‐economic and demographic inequalities, depending on the respective outcome variable (Table 1). In the regressions of individual barriers/facilitators, only those participants were included who had the respective barrier/facilitator applicable to them. *p* values ≤ 0.05 were considered statistically significant.

Sensitivity analyses were conducted for the overall barrier/facilitator burden. Not all investigated barriers/facilitators were applicable to all participants; however, the calculated overall barrier/facilitator scores do not account for this point. Therefore, in the sensitivity analyses, the percentages of encountered major barriers/very helpful facilitators out of the respective applicable ones were additionally calculated and used as outcome variables. Furthermore, we conducted sensitivity analyses for the individual barriers and facilitators, in which we grouped ‘not applicable’ and ‘no problem’ together. Moreover, all analyses were repeated, excluding respondents with a completion time below 10 min, assuming that these are potentially hasty completions.

Data analysis was conducted using Stata 18 (StataCorp LLC, Texas, the United States). The analysis included all participants who completed the full survey based on a complete case analysis. The study was conducted and reported in accordance with the STROBE (Strengthening the Reporting of Observational Studies in Epidemiology) checklist for cross‐sectional studies [52], which is presented in Table SA1.

## 3 Results

### 3.1 Sample Characteristics

Overall, 558 people started the survey. Out of these, 70 indicated that they did not fulfil the eligibility criteria, and 55 did not complete the full survey, resulting in the final sample of 433 LC patients. No differences in sample characteristics were observed between participants who dropped out and those who finished the survey. Average completion time was 18.2 min (SD 6.6) and 33 (7.6%) participants finished in less than 10 min.

Of the 433 participants, 84.1% (*n* = 364) were women, the mean age was 43.0 years (SD 11.0), 52.7% (*n* = 228) resided in an urban area, and 25.6% (*n* = 111) had a complementary private health insurance. Most participants reported LC onset in 2022 (43.2%), the mean duration of LC was 29.0 months (SD 12.1), with 400 (92.4%) individuals with ongoing LC at the time of the study compared to 33 (7.6%) who considered themselves recovered. The mean number of perceived severe symptoms was 5.9 (SD 2.4) out of 11. Detailed sample characteristics are presented in Table 2.

**Table 2:** Sample characteristics (*n* = 433).

	Frequency (%)		Frequency (%)
Gender		Urbanicity	
Female	364 (84.1%)	Urban	228 (52.7%)
Male	66 (15.2%)	Rural	205 (47.3%)
Diverse	1 (0.2%)	Health‐related background	
Do not want to answer	2 (0.5%)	No health‐related background	299 (69.1%)
Age		Health‐related background	134 (30.9%)
18‐30	67 (15.5%)	Education	
31‐40	118 (27.3%)	Lower than A‐levels	111 (26.1%)
41‐50	124 (28.6%)	A‐levels	70 (16.5%)
51‐60	108 (24.9%)	Higher than A‐levels	244 (57.4%)
older than 60	16 (3.7%)	Other	8 (1.8%)
Federal state		Central co‐ordination	
Burgenland	17 (3.9%)	No central co‐ordination available	322 (74.4%)
Carinthia	10 (2.3%)	Central co‐ordination available	111 (25.6%)
Lower Austria	57 (13.2%)	Household economic situation during LC	
Upper Austria	51 (11.8%)	Good or very good	91 (21.2%)
Salzburg	19 (4.4%)	Medium	162 (37.7%)
Styria	52 (12.0%)	Bad or very bad	177 (41.2%)
Tyrol	92 (21.2%)	Prior physical chronic conditions	
Vorarlberg	19 (4.4%)	No chronic conditions	288 (67.1%)
Vienna	116 (26.8%)	Prior chronic condition(s)	141 (32.9%)
Migration background		Prior mental health conditions	
No migration background	382 (88.2%)	No mental health issues	331 (78.4%)
Migration background	49 (11.3%)	Prior mental health issues	91 (21.6%)
Do not want to answer	2 (0.5%)	Number of severe LC symptoms (SD)	5.86 (2.39)
Complementary private health insurance		Year of LC onset	
No complementary health insurance	319 (74.2%)	2020	48 (11.2%)
Complementary health insurance	111 (25.8%)	2021	95 (22.2%)
Relationship status		2022	187 (43.8%)
Alone	133 (30.7%)	2023	74 (17.3%)
In a relationship	300 (69.3%)	2024	23 (5.4%)
Total	433	LC duration at participation in months (SD)	29.0 (12.1)
		Total	433

The descriptive results for each assessed barrier and facilitator are shown in Tables 3 and 4.

**Table 3:** Healthcare access barriers by ‘access to care’ dimension (Levesque et al. 2013) for adult long Covid‐19 patients in Austria (*n* = 433).

	Barrier	Not applicable	No problems	Minor problems	Major problems
*Approachability*	Too little available information about the condition and treatment options	1%	5%	24%	70%
	Difficulties in assessing the credibility of available information	1%	17%	41%	40%
	Difficulties in perceiving symptoms as unusual and in attributing them to the condition	1%	16%	34%	49%
*Acceptability*	GPs did not take me and my condition seriously	2%	22%	28%	48%
	Specialists did not take me and my condition seriously	2%	15%	32%	51%
	General practitioners attributed my symptoms to mental health problems (e.g., depression and psychosomatic complaints)	8%	30%	24%	37%
	Specialists attributed my symptoms to mental health problems (e.g., depression and psychosomatic complaints)	5%	20%	36%	39%
	Doctors or assessors ignored the findings of other medical staff or did not take them seriously	16%	11%	21%	52%
	Friends or family did not take my condition seriously	1%	23%	42%	33%
	My professional environment did not take my condition seriously	9%	24%	32%	35%
	I questioned myself as to whether I was really ill	5%	26%	37%	32%
	I did not know my way around the health system and did not know what to look for	12%	27%	38%	24%
*Availability and accommodation*	Outpatient clinics or resident specialists required a referral from other doctors or certain diagnoses	9%	26%	35%	30%
	I had to see doctors more often than necessary to provide my health insurance provider with ongoing evidence of my condition	14%	16%	23%	47%
	GPs did not accept new patients or only had open appointments in the distant future	19%	54%	15%	13%
	Resident specialists did not accept new patients or only had open appointments in the distant future	5%	14%	25%	56%
	Hospital outpatient clinics, incl. long Covid‐19 clinics, did not accept new patients or only had distant‐future appointments	23%	9%	17%	51%
	GPs didn't take enough time for me	5%	30%	28%	36%
	Specialists didn't take enough time for me	4%	25%	43%	28%
	Too little time was taken for me in the hospital outpatient clinic	45%	14%	18%	24%
	Long Covid‐19 outpatient clinics were closed before my treatment was completed/started	50%	13%	6%	31%
	The facilities in the waiting areas of the doctors I visited did not meet my needs (e.g., too loud and too bright)	8%	15%	33%	44%
	Travelling to my GP was a burden (e.g., far away and poor public transport options)	8%	31%	30%	31%
	Travelling to relevant specialists was a burden (e.g., far away and poor public transport options)	3%	12%	29%	56%
	Travelling to relevant hospital outpatient clinics was a burden (e.g., far away and poor public transport options)	37%	12%	14%	37%
	Telemedicine or home visits were not offered by GPs, although I would have needed them	36%	15%	15%	33%
	Telemedicine or home visits were not offered by specialists, although I would have needed them	35%	17%	19%	29%
	Organising my treatments or administrative procedures myself was a burden (e.g., researching services and making appointments)	2%	5%	20%	73%
*Affordability*	Costs for GPs posed a financial burden for me	12%	56%	21%	11%
	Costs for specialists posed a financial burden for me	5%	12%	32%	51%
	Costs for medication posed a financial burden for me	5%	15%	37%	42%
	Costs for dietary supplements posed a financial burden for me	6%	9%	27%	58%
	Costs for other treatments (e.g., physiotherapy, psychotherapy and homoeopathy) posed a financial burden for me	7%	9%	25%	59%
	I had to weigh up different treatments against each other due to costs	9%	12%	26%	52%
	I had to go to private doctors as I could not find statutory insurance‐covered doctors with long Covid‐19 expertise	10%	5%	12%	74%
	I had to go to elective or private doctors due to waiting times at doctors covered by statutory health insurance	20%	10%	21%	49%
*Appropriateness*	My GP had difficulties diagnosing long Covid‐19	5%	22%	21%	51%
	The specialists I consulted had difficulties diagnosing long Covid‐19	2%	28%	33%	36%
	My GP had difficulties treating my symptoms	4%	7%	15%	74%
	The specialists I consulted had difficulties treating my symptoms	3%	12%	30%	56%
	I had difficulties receiving or extending sick pay	23%	21%	24%	32%
	I had difficulties obtaining a state disability pension	50%	2%	5%	43%
	I had difficulties being allowed to work part‐time during reintegration	64%	14%	5%	18%
	Friends and family had difficulties adjusting to my long Covid‐19 limitations	1%	10%	45%	44%
	My professional environment had difficulties adapting to my long Covid‐19 limitations	23%	8%	28%	41%
	There is no diagnostic test/biomarker via, for example, a blood count that enables a clear, objective long Covid‐19 diagnosis*	4%	7%	18%	71%
	I had difficulties obtaining a private disability pension	85%	1%	1%	13%

* *n* = 432 for this barrier, as one person did not answer this question.

**Table 4:** Healthcare access facilitators for adult long Covid‐19 patients in Austria (*n* = 433).

Facilitator	Not applicable	Not helpful	Somewhat helpful	Very helpful
My social environment and family were a great support to me	2%	9%	28%	62%
My professional environment was very understanding	10%	25%	43%	22%
The Long Covid Austria Facebook group provided useful information	27%	5%	28%	40%
The exchange with other patients helped me	7%	5%	36%	52%
My GP coordinated my treatment	28%	46%	20%	6%
GPs encouraged me in the perception of my illness	19%	40%	25%	16%
Specialists encouraged me in the perception of my illness	10%	18%	36%	37%
Doctors offered to shorten waiting times by switching to a private practice	74%	14%	7%	5%
Doctors offered to shorten waiting times through private payments	82%	11%	4%	3%
Telemedicine facilitated my treatment	55%	11%	15%	20%

### 3.2 Barriers

The mean number of barriers perceived as minor or major problem was 31.9 (SD 8.4) out of 47, and the mean barrier score was 52.0 (SD 17.1) out of 94. We found that LC patients living in rural areas and patients with a poor/very poor household economic situation had significantly higher overall barrier scores. By contrast, patients with complementary private health insurance or with a good/very good household economic situation had lower overall barrier scores. Detailed results are presented in Tables 5 and SA2.

**Table 5:** Regression results for overall barrier and facilitator scores, as well as unmet healthcare needs.

*Independent variables*	*Overall barrier score coefficient [95% CI]*	*Overall facilitator score coefficient [95% CI]*	*Overall unmet healthcare needs odds ratio [95% CI]*
Female gender	−1.632 [−5.416, 2.152]	−0.064 [−0.909, 0.78]	0.796 [0.400, 1.584]
*Aged 18–30 (base case)*			
Aged 31–40	0.577 [−3.854, 5.008]	−0.280 [−1.269, 0.709]	0.568 [0.251, 1.285]
Aged 41–50	0.230 [−4.069, 4.528]	−0.342 [−1.301, 0.618]	0.852 [0.381, 1.905]
Aged 51–60	−2.015 [−6.615, 2.584]	−0.716 [−1.743, 0.31]	0.658 [0.279, 1.549]
Older than 60	−7.605 [−15.87, 0.66]	1.642 [−0.202, 3.487]	0.548 [0.134, 2.244]
Living in a rural area	3.005* [0.061, 5.948]	0.394 [−0.263, 1.051]	1.786* [1.032, 3.091]
Health‐related background	−1.680 [−4.678, 1.318]	−0.067 [−0.736, 0.602]	1.366 [0.780, 2.39]
Complementary health insurance	−3.233* [−6.411, −0.054]	0.375 [−0.334, 1.084]	0.933 [0.527, 1.651]
The household economic situation is good or very good	−11.090*** [−14.854, −7.326]	−0.412 [−1.252, 0.428]	0.538 [0.287, 1.008]
*Household economic situation medium (base case)*			
The household economic situation is bad or very bad	5.871*** [2.715, 9.028]	−0.139 [−0.843, 0.566]	1.473 [0.809, 2.684]
*N*	387	387	387

*Note:* Red numbers refer to adverse patient experiences, green to beneficial ones. Control variables included: Living in a federal state with central co‐ordination services, education, migration background, relationship status, prior physical or mental health conditions, number of perceived severe long Covid‐19 (LC) symptoms, year of LC onset and LC duration. **p value < 0.01

* *p* value < 0.05

** *p* value < 0.001.

On an individual barrier level, we found inequalities along all dimensions of the ‘access to care’ framework [32]. Some variables of interest showed consistent association directions across barriers (urbanicity, complementary private health insurance and economic situation), while for other variables, the direction was dependent on the individual barrier (age and health‐related background).

In line with the findings on the overall barrier score, living in a rural area or having a disadvantaged economic situation increased the odds of perceiving individual barriers as more problematic, whereas having complementary insurance or an advantaged economic situation reduced them. We find urbanicity to be statistically significantly associated with six barriers, particularly regarding the dimension *Approachability*, including available information, and *Availability and accommodation*, including the burden to travel to specialists and hospital outpatient clinics. Notably, travelling to GPs was not affected by urbanicity.

Having complementary health insurance was a statistically significant factor in reducing the burden for 10 barriers, mainly related to specialist care, including not being taken seriously, burdensome costs, difficulties in diagnosing and treating, and having to resort to private (non‐contracted) specialists due to a lack of expertise in the public sector. However, having complementary private health insurance was also significantly associated with GPs having difficulties in diagnosing LC and treating symptoms.

Household economic situation proved to be the most prominent association and was statistically significant for 29 out of 47 barriers. Patients' economic situation was statistically significantly associated with barriers regarding all levels of care and particularly GPs. For GP care, associated barriers included not being taken seriously, attribution of symptoms to mental health conditions, burdensome costs, difficulties diagnosing and treating LC, travel to the practice, and availability of telemedicine/home visits. For specialist care, burdensome costs and insufficient consultation duration were associated with the economic situation. Ultimately, for hospital care, consultation duration was statistically significant, as was closure of LC outpatient clinics prior to completion/start of treatment.

Higher age was statistically significantly associated with perceiving four barriers as less problematic and five barriers as more problematic. Older participants were less likely to report difficulties travelling to a GP or specialists' difficulties with symptom treatment. However, they were more likely to report GP and specialist difficulties in diagnosing LC and not being taken seriously in a professional environment.

A health‐related background was advantageous regarding four barriers, but also problematic in two. Among others, not knowing one's way around the health system was significantly less of a burden to those with a health‐related background, as were adjustment difficulties by friends and family. In a professional context, however, it increased participants' problems regarding adjustment difficulties and being allowed to work part‐time. Detailed results are depicted in Tables 6 and SA3.

**Table 6:** Regression results for individual barriers by ‘access to care’ dimension (Levesque et al. 2013) for adult long Covid‐19 patients in Austria.

	*Approachability*			*Acceptability (I/II)*			
	Too little available information about the condition and treatment options	Difficulties in assessing the credibility of available information	Difficulties in perceiving symptoms as unusual and in attributing them	GPs did not take me and my condition seriously	Specialists did not take me and my condition seriously	GPs attributed my symptoms to mental health problems	Specialists attributed my symptoms to mental health problems
*Independent variables*	*Odds ratio [95% CI]*	*Odds ratio [95% CI]*	*Odds ratio [95% CI]*	*Odds ratio [95% CI]*	*Odds ratio [95% CI]*	*Odds ratio [95% CI]*	*Odds ratio [95% CI]*
Female gender	0.588 [0.288, 1.200]	1.179 [0.687, 2.022]	1.110 [0.642, 1.920]	1.076 [0.609, 1.898]	0.997 [0.575, 1.728]	1.017 [0.576, 1.798]	0.991 [0.567, 1.733]
*Aged 18–30 (base case)*							
Aged 31–40	0.770 [0.366, 1.619]	1.076 [0.571, 2.031]	0.975 [0.510, 1.862]	0.929 [0.495, 1.745]	0.666 [0.341, 1.301]	0.855 [0.453, 1.617]	0.911 [0.478, 1.738]
Aged 41–50	1.063 [0.512, 2.208]	1.335 [0.721, 2.472]	0.748 [0.402, 1.392]	0.905 [0.489, 1.673]	0.530 [0.273, 1.032]	1.023 [0.551, 1.900]	0.661 [0.351, 1.246]
Aged 51–60	1.227 [0.550, 2.738]	1.759 [0.899, 3.442]	1.303 [0.664, 2.557]	0.981 [0.502, 1.919]	0.790 [0.384, 1.624]	0.775 [0.393, 1.525]	1.026 [0.518, 2.031]
Older than 60	0.803 [0.216, 2.985]	2.989 [0.847, 10.553]	1.315 [0.380, 4.546]	0.825 [0.255, 2.669]	0.460 [0.128, 1.653]	0.611 [0.191, 1.953]	1.144 [0.320, 4.090]
Living in a rural area	1.907* [1.142, 3.183]	2.257*** [1.470, 3.466]	1.018 [0.669, 1.550]	1.128 [0.734, 1.735]	0.963 [0.618, 1.501]	1.084 [0.699, 1.680]	1.105 [0.717, 1.703]
Health‐related background	0.805 [0.481, 1.346]	0.795 [0.510, 1.240]	0.784 [0.511, 1.202]	0.825 [0.533, 1.275]	1.367 [0.872, 2.143]	0.822 [0.526, 1.285]	1.151 [0.748, 1.77]
Complementary health insurance	0.535* [0.317, 0.903]	0.986 [0.624, 1.559]	0.955 [0.603, 1.513]	0.711 [0.449, 1.126]	0.608* [0.376, 0.981]	0.697 [0.431, 1.128]	0.713 [0.446, 1.138]
The household economic situation is good or very good	0.665 [0.362, 1.224]	0.499* [0.287, 0.869]	0.884 [0.518, 1.508]	0.297*** [0.173, 0.509]	0.594 [0.344, 1.026]	0.564* [0.323, 0.983]	0.911 [0.527, 1.574]
*Household economic situation medium (base case)*							
The household economic situation is bad or very bad	1.992* [1.124, 3.533]	1.268 [0.802, 2.004]	1.41 [0.895, 2.221]	1.081 [0.679, 1.722]	1.150 [0.712, 1.858]	1.445 [0.906, 2.304]	1.477 [0.929, 2.349]
*N*	384	383	381	379	380	354	366

	*Acceptability (II/II)*					*Availability and accommodation (I/III)*	
	Doctors or assessors ignored the findings of other medical staff or did not take them seriously	Friends or family did not take my condition seriously	My professional environment did not take my condition seriously	I questioned myself as to whether I was really ill	I did not know my way around the health system and did not know what to look for	Outpatient clinics or resident specialists required a referral from other doctors or certain diagnoses	I had to see doctors more often than necessary to provide my health insurance with ongoing evidence of my condition
*Independent variables*	*Odds ratio [95% CI]*	*Odds ratio [95% CI]*	*Odds ratio [95% CI]*	*Odds ratio [95% CI]*	*Odds ratio [95% CI]*	*Odds ratio [95% CI]*	*Odds ratio [95% CI]*
Female gender	1.001 [0.508, 1.972]	0.667 [0.387, 1.150]	0.968 [0.552, 1.700]	0.990 [0.594, 1.649]	1.465 [0.825, 2.602]	0.987 [0.578, 1.688]	0.926 [0.513, 1.670]
*Aged 18–30 (base case)*							
Aged 31–40	1.585 [0.735, 3.416]	0.776 [0.418, 1.438]	1.092 [0.570, 2.093]	1.544 [0.817, 2.917]	0.838 [0.427, 1.647]	1.083 [0.559, 2.098]	1.883 [0.924, 3.838]
Aged 41–50	1.895 [0.918, 3.911]	1.164 [0.647, 2.091]	1.812 [0.955, 3.437]	0.898 [0.488, 1.654]	0.953 [0.498, 1.824]	1.304 [0.682, 2.496]	1.703 [0.851, 3.407]
Aged 51–60	1.491 [0.671, 3.314]	1.673 [0.883, 3.170]	2.515** [1.271, 4.978]	1.017 [0.515, 2.006]	0.838 [0.413, 1.701]	1.342 [0.666, 2.703]	1.581 [0.742, 3.369]
Older than 60	2.856 [0.517, 15.78]	0.809 [0.245, 2.665]	1.186 [0.236, 5.951]	0.529 [0.180, 1.554]	0.745 [0.219, 2.536]	0.929 [0.275, 3.133]	1.370 [0.300, 6.260]
Living in a rural area	0.905 [0.530, 1.546]	1.347 [0.890, 2.039]	1.255 [0.812, 1.939]	1.199 [0.787, 1.826]	1.603* [1.009, 2.549]	0.877 [0.569, 1.353]	1.320 [0.820, 2.125]
Health‐related background	1.130 [0.652, 1.957]	0.805 [0.526, 1.233]	1.452 [0.927, 2.274]	0.850 [0.554, 1.306]	0.200*** [0.12, 0.332]	0.903 [0.581, 1.403]	0.862 [0.529, 1.407]
Complementary health insurance	0.971 [0.557, 1.692]	0.824 [0.528, 1.287]	0.988 [0.617, 1.582]	0.829 [0.523, 1.315]	0.419** [0.249, 0.704]	0.695 [0.436, 1.109]	0.523* [0.315, 0.867]
The household economic situation is good or very good	0.336*** [0.177, 0.639]	0.650 [0.379, 1.114]	0.417** [0.239, 0.728]	1.147 [0.677, 1.942]	0.599 [0.335, 1.071]	0.730 [0.418, 1.278]	0.515* [0.279, 0.953]
*Household economic situation medium (base case)*							
The household economic situation is bad or very bad	2.546** [1.438, 4.51]	1.173 [0.751, 1.833]	0.91 [0.569, 1.456]	1.313 [0.828, 2.082]	1.158 [0.715, 1.878]	1.47 [0.926, 2.332]	1.403 [0.844, 2.333]
*N*	325	383	353	368	339	354	334

	*Availability and accommodation (II/III)*						
	GPs did not accept new patients or only had open appointments in the distant future	Resident specialists did not accept new patients or only had open appointments in the distant future	Hospital outpatient clinics incl. long Covid‐19 clinics did not accept new patients or only had distant‐future appointments	GPs didn't take enough time for me	Specialists didn't take enough time for me	Too little time was taken for me in the hospital outpatient clinic	Long Covid‐19 outpatient clinics were closed before my treatment was completed/started
*Independent variables*	*Odds ratio [95% CI]*	*Odds ratio [95% CI]*	*Odds ratio [95% CI]*	*Odds ratio [95% CI]*	*Odds ratio [95% CI]*	*Odds ratio [95% CI]*	*Odds ratio [95% CI]*
Female gender	0.704 [0.373, 1.326]	1.108 [0.615, 1.997]	1.103 [0.566, 2.149]	1.605 [0.912, 2.823]	1.146 [0.663, 1.982]	0.649 [0.324, 1.301]	1.024 [0.451, 2.327]
*Aged 18–30 (base case)*							
Aged 31–40	1.375 [0.614, 3.079]	0.909 [0.432, 1.914]	1.475 [0.656, 3.313]	0.809 [0.437, 1.497]	1.003 [0.533, 1.886]	0.746 [0.321, 1.736]	1.069 [0.407, 2.808]
Aged 41–50	1.860 [0.863, 4.011]	0.643 [0.312, 1.323]	1.151 [0.541, 2.447]	0.860 [0.475, 1.558]	0.707 [0.380, 1.314]	0.863 [0.375, 1.984]	1.698 [0.634, 4.545]
Aged 51–60	1.283 [0.548, 2.999]	0.534 [0.250, 1.141]	0.984 [0.423, 2.289]	1.035 [0.541, 1.982]	0.968 [0.490, 1.914]	0.740 [0.296, 1.850]	1.100 [0.379, 3.187]
Older than 60	0.985 [0.172, 5.634]	0.546 [0.152, 1.960]	1.936 [0.306, 12.242]	0.532 [0.160, 1.773]	0.706 [0.205, 2.432]	3.512 [0.489, 25.249]	0.981 [0.138, 6.986]
Living in a rural area	0.697 [0.413, 1.176]	0.771 [0.486, 1.223]	1.306 [0.753, 2.268]	1.264 [0.831, 1.921]	0.877 [0.574, 1.339]	0.965 [0.556, 1.675]	0.963 [0.484, 1.917]
Health‐related background	0.518* [0.295, 0.910]	0.807 [0.505, 1.290]	1.072 [0.602, 1.907]	0.815 [0.531, 1.252]	1.337 [0.869, 2.056]	0.871 [0.489, 1.550]	0.849 [0.416, 1.734]
Complementary health insurance	0.709 [0.398, 1.265]	0.430*** [0.263, 0.704]	0.705 [0.394, 1.259]	0.857 [0.541, 1.357]	0.722 [0.458, 1.138]	0.807 [0.453, 1.437]	1.024 [0.472, 2.223]
The household economic situation is good or very good	0.752 [0.359, 1.572]	0.588 [0.328, 1.055]	0.663 [0.329, 1.333]	0.434** [0.252, 0.745]	0.544* [0.315, 0.940]	0.387* [0.181, 0.829]	0.308* [0.126, 0.755]
*Household economic situation medium (base case)*							
The household economic situation is bad or very bad	1.574 [0.901, 2.748]	0.912 [0.548, 1.519]	1.886* [1.048, 3.395]	1.287 [0.82, 2.022]	1.44 [0.916, 2.266]	1.073 [0.592, 1.945]	1.166 [0.532, 2.556]
*N*	319	370	303	369	371	217	200

	*Availability and accommodation (III/III)*						
	The facilities in the waiting areas of the doctors I visited did not meet my needs (e.g., too loud, too bright)	Travelling to my GP was a burden (e.g., far away, poor public transport options)	Travelling to relevant specialists was a burden (e.g., far away, poor public transport options)	Travelling to relevant hospital outpatient clinics was a burden (e.g., far away, public transport options)	Telemedicine or home visits were not offered by GPs, although I would have needed them	Telemedicine or home visits were not offered by specialists, although I would have needed them	Organising my treatments or administrative procedures myself was a burden (e.g., making appointments)
*Independent variables*	*Odds ratio [95% CI]*	*Odds ratio [95% CI]*	*Odds ratio [95% CI]*	*Odds ratio [95% CI]*	*Odds ratio [95% CI]*	*Odds ratio [95% CI]*	*Odds ratio [95% CI]*
Female gender	2.078* [1.176, 3.672]	1.127 [0.648, 1.958]	1.554 [0.876, 2.758]	0.960 [0.468, 1.966]	0.658 [0.327, 1.323]	0.699 [0.348, 1.405]	0.833 [0.418, 1.661]
*Aged 18–30 (base case)*							
Aged 31–40	0.728 [0.365, 1.449]	0.905 [0.475, 1.724]	1.039 [0.509, 2.121]	0.823 [0.346, 1.959]	1.747 [0.793, 3.848]	1.157 [0.545, 2.457]	1.308 [0.613, 2.791]
Aged 41–50	0.849 [0.427, 1.686]	0.889 [0.469, 1.684]	0.662 [0.331, 1.324]	1.181 [0.497, 2.81]	1.422 [0.674, 3.000]	1.033 [0.507, 2.104]	1.928 [0.903, 4.116]
Aged 51–60	0.571 [0.279, 1.168]	0.449* [0.225, 0.898]	0.556 [0.267, 1.159]	0.785 [0.3, 2.054]	0.878 [0.385, 2.003]	0.582 [0.263, 1.290]	1.171 [0.537, 2.552]
Older than 60	0.238* [0.065, 0.878]	1.273 [0.372, 4.362]	0.385 [0.12, 1.234]	0.254 [0.039, 1.669]	1.549 [0.295, 8.122]	0.52 [0.082, 3.297]	0.520 [0.132, 2.043]
Living in a rural area	1.211 [0.775, 1.894]	1.362 [0.884, 2.099]	2.256*** [1.408, 3.613]	2.881*** [1.588, 5.225]	1.537 [0.89, 2.652]	1.606 [0.954, 2.703]	1.547 [0.907, 2.640]
Health‐related background	0.792 [0.504, 1.246]	0.985 [0.632, 1.536]	0.856 [0.533, 1.374]	0.923 [0.512, 1.666]	0.933 [0.531, 1.637]	0.766 [0.449, 1.305]	0.816 [0.475, 1.399]
Complementary health insurance	1.153 [0.708, 1.878]	0.954 [0.594, 1.533]	0.736 [0.451, 1.202]	0.687 [0.374, 1.262]	0.843 [0.472, 1.505]	0.583 [0.337, 1.008]	0.680 [0.390, 1.185]
The household economic situation is good or very good	0.490* [0.278, 0.862]	0.437** [0.247, 0.773]	0.685 [0.388, 1.211]	0.700 [0.329, 1.488]	0.455* [0.218, 0.953]	0.577 [0.281, 1.185]	0.494* [0.264, 0.925]
*Household economic situation medium (base case)*							
The household economic situation is bad or very bad	1.486 [0.914, 2.414]	1.178 [0.736, 1.885]	1.213 [0.727, 2.025]	1.301 [0.685, 2.472]	1.349 [0.768, 2.369]	1.223 [0.707, 2.115]	1.279 [0.71, 2.304]
*N*	362	358	374	248	254	255	378

	*Affordability (I/II)*						
	Costs for GPs posed a financial burden for me	Costs for specialists posed a financial burden for me	Costs for medication posed a financial burden for me	Costs for dietary supplements posed a financial burden for me	Costs for other treatments (e.g., physiotherapy and homoeopathy) posed a financial burden for me	I had to weigh up different treatments against each other due to costs	I had to go to private doctors as I could not find insurance‐covered doctors with long Covid‐19 expertise
*Independent variables*	*Odds ratio [95% CI]*	*Odds ratio [95% CI]*	*Odds ratio [95% CI]*	*Odds ratio [95% CI]*	*Odds ratio [95% CI]*	*Odds ratio [95% CI]*	*Odds ratio [95% CI]*
Female gender	0.557 [0.3, 1.033]	0.706 [0.386, 1.29]	0.743 [0.409, 1.351]	1.315 [0.684, 2.528]	1.418 [0.73, 2.755]	1.289 [0.682, 2.436]	1.382 [0.601, 3.179]
*Aged 18–30 (base case)*							
Aged 31–40	1.074 [0.503, 2.293]	0.731 [0.356, 1.504]	1.111 [0.550, 2.241]	1.567 [0.709, 3.465]	1.24 [0.574, 2.678]	1.876 [0.891, 3.95]	0.538 [0.187, 1.546]
Aged 41–50	1.556 [0.749, 3.233]	0.736 [0.365, 1.483]	0.811 [0.411, 1.599]	1.497 [0.704, 3.182]	1.619 [0.766, 3.425]	1.612 [0.796, 3.266]	1.205 [0.400, 3.630]
Aged 51–60	0.824 [0.368, 1.845]	0.485 [0.230, 1.024]	0.784 [0.379, 1.621]	0.930 [0.416, 2.081]	0.928 [0.412, 2.090]	1.559 [0.702, 3.462]	0.406 [0.138, 1.198]
Older than 60	0.151 [0.016, 1.418]	0.502 [0.137, 1.847]	0.472 [0.140, 1.591]	1.171 [0.276, 4.968]	0.367 [0.104, 1.289]	0.161* [0.040, 0.651]	0.432 [0.082, 2.267]
Living in a rural area	1.495 [0.901, 2.481]	1.655* [1.04, 2.633]	1.414 [0.897, 2.230]	1.573 [0.927, 2.668]	1.245 [0.733, 2.116]	1.187 [0.719, 1.958]	0.988 [0.505, 1.935]
Health‐related background	0.796 [0.475, 1.334]	0.719 [0.448, 1.153]	0.936 [0.590, 1.487]	0.978 [0.569, 1.679]	0.581* [0.338, 0.999]	0.697 [0.412, 1.181]	0.591 [0.295, 1.184]
Complementary health insurance	0.788 [0.448, 1.384]	0.502** [0.307, 0.821]	1.065 [0.649, 1.748]	1.310 [0.743, 2.308]	0.739 [0.428, 1.275]	0.632 [0.376, 1.060]	0.307*** [0.155, 0.607]
The household economic situation is good or very good	0.488 [0.229, 1.042]	0.293*** [0.164, 0.524]	0.365*** [0.204, 0.654]	0.266*** [0.144, 0.491]	0.313*** [0.171, 0.573]	0.252*** [0.136, 0.468]	0.290** [0.135, 0.620]
*Household economic situation medium (base case)*							
The household economic situation is bad or very bad	1.849* [1.091, 3.132]	2.812*** [1.683, 4.698]	3.313*** [2.003, 5.479]	5.503*** [2.948, 10.271]	3.787*** [2.042, 7.021]	3.348*** [1.893, 5.921]	2.803* [1.215, 6.463]
*N*	343	369	366	365	360	352	349

	*Affordability (II/II)*	*Appropriateness (I/II)*					
	I had to go to elective or private doctors due to waiting times at doctors covered by statutory health insurance	My GP had difficulties diagnosing long Covid‐19	The specialists I consulted had difficulties diagnosing long Covid‐19	My GP had difficulties treating my symptoms	The specialists I consulted had difficulties treating my symptoms	I had difficulties receiving or extending sick pay	I had difficulties obtaining a state disability pension
*Independent variables*	*Odds ratio [95% CI]*	*Odds ratio [95% CI]*	*Odds ratio [95% CI]*	*Odds ratio [95% CI]*	*Odds ratio [95% CI]*	*Odds ratio [95% CI]*	*Odds ratio [95% CI]*
Female gender	1.169 [0.614, 2.229]	0.958 [0.530, 1.731]	0.903 [0.528, 1.543]	1.376 [0.683, 2.774]	0.970 [0.550, 1.712]	1.133 [0.614, 2.090]	0.767 [0.189, 3.106]
*Aged 18–30 (base case)*							
Aged 31–40	1.417 [0.662, 3.034]	1.982* [1.031, 3.81]	1.261 [0.664, 2.397]	1.196 [0.486, 2.944]	0.523 [0.257, 1.068]	0.901 [0.416, 1.953]	1.16 [0.172, 7.805]
Aged 41–50	1.522 [0.726, 3.189]	1.752 [0.936, 3.281]	1.876* [1.002, 3.514]	1.002 [0.437, 2.297]	0.648 [0.325, 1.290]	0.843 [0.398, 1.788]	1.592 [0.221, 11.468]
Aged 51–60	1.181 [0.524, 2.661]	2.055* [1.028, 4.107]	1.514 [0.776, 2.953]	0.558 [0.234, 1.328]	0.424* [0.203, 0.884]	0.907 [0.408, 2.015]	1.101 [0.156, 7.785]
Older than 60	0.975 [0.241, 3.940]	3.000 [0.896, 10.042]	1.966 [0.584, 6.617]	0.536 [0.132, 2.178]	0.709 [0.179, 2.802]	*n/a*	*n/a*
Living in a rural area	1.402 [0.825, 2.380]	0.962 [0.615, 1.504]	1.012 [0.671, 1.528]	1.131 [0.641, 1.995]	1.050 [0.670, 1.646]	0.994 [0.610, 1.62]	0.81 [0.224, 2.932]
Health‐related background	0.820 [0.478, 1.407]	0.799 [0.506, 1.261]	1.023 [0.674, 1.551]	0.753 [0.421, 1.35]	0.990 [0.632, 1.550]	0.915 [0.559, 1.499]	1.417 [0.441, 4.551]
Complementary health insurance	0.581 [0.335, 1.010]	0.614* [0.380, 0.990]	0.608* [0.385, 0.960]	0.391** [0.217, 0.707]	0.669 [0.412, 1.086]	0.750 [0.433, 1.300]	0.794 [0.241, 2.621]
The household economic situation is good or very good	0.441* [0.231, 0.843]	0.394** [0.226, 0.688]	0.977 [0.573, 1.665]	0.322** [0.161, 0.646]	0.643 [0.365, 1.135]	0.426* [0.211, 0.86]	0.412 [0.115, 1.471]
*Household economic situation medium (base case)*							
The household economic situation is bad or very bad	1.217 [0.678, 2.183]	0.894 [0.546, 1.462]	0.982 [0.627, 1.538]	0.826 [0.429, 1.594]	0.904 [0.556, 1.470]	2.840*** [1.71, 4.719]	7.593** [1.733, 33.275]

	*Appropriateness (II/II)*				
	I had difficulties being allowed to work part‐time during reintegration	Friends and family had difficulties adjusting to my long Covid‐19 limitations	My professional environment had difficulties adapting to my long Covid‐19 limitations	There is no diagnostic test/biomarker via, for example, a blood count that enables a clear, objective long Covid‐19 diagnosis	I had difficulties obtaining a private disability pension
*Independent variables*	*Odds ratio [95% CI]*	*Odds ratio [95% CI]*	*Odds ratio [95% CI]*	*Odds ratio [95% CI]*	*Odds ratio [95% CI]*
Female gender	1.357 [0.483, 3.809]	0.633 [0.357, 1.120]	0.995 [0.526, 1.884]	0.597 [0.284, 1.256]	*Insufficient number of participants to whom this barrier was applicable*
*Aged 18–30 (base case)*					
Aged 31–40	0.599 [0.148, 2.423]	1.036 [0.541, 1.986]	0.931 [0.437, 1.984]	1.694 [0.795, 3.606]	
Aged 41–50	0.374 [0.096, 1.453]	0.892 [0.472, 1.686]	0.988 [0.478, 2.041]	1.818 [0.865, 3.820]	
Aged 51–60	0.673 [0.159, 2.843]	1.303 [0.661, 2.571]	1.576 [0.704, 3.525]	2.882* [1.245, 6.671]	
Older than 60	*n/a*	0.902 [0.270, 3.016]	0.417 [0.047, 3.714]	0.901 [0.238, 3.405]	
Living in a rural area	1.571 [0.743, 3.325]	1.341 [0.870, 2.065]	1.121 [0.685, 1.836]	1.278 [0.746, 2.188]	
Health‐related background	2.672* [1.205, 5.926]	0.633* [0.407, 0.984]	1.887* [1.099, 3.24]	0.663 [0.387, 1.137]	
Complementary health insurance	1.158 [0.496, 2.703]	1.411 [0.884, 2.252]	1.056 [0.617, 1.806]	0.632 [0.360, 1.111]	
The household economic situation is good or very good	0.798 [0.292, 2.179]	0.719 [0.412, 1.252]	0.377** [0.201, 0.707]	0.802 [0.413, 1.557]	
*Household economic situation medium (base case)*					
The household economic situation is bad or very bad	1.885 [0.840, 4.231]	1.503 [0.942, 2.399]	1.088 [0.624, 1.899]	0.903 [0.500, 1.633]	
*N*	141	382	297	373	58

*Note:* Red numbers refer to adverse patient experiences, green to beneficial ones. Control variables included: Living in a federal state with central co‐ordination services, education, migration background, relationship status, prior physical or mental health conditions, number of perceived severe long Covid‐19 (LC) symptoms, year of LC onset, and LC duration.

* *p* value < 0.05

** *p* value < 0.01

*** *p* value < 0.001.

Results of all conducted sensitivity analyses were in line with the main analysis presented above.

### 3.3 Facilitators

The mean facilitator score was 7.6 (SD 3.0) out of 20. We did not find any statistically significant differences by socio‐economic and demographic characteristics in the overall facilitator score. By contrast, there were differences when looking at individual facilitators. Individuals aged 51–60, that is, being in a working age but close to retirement, were, among others, less likely to perceive their social environment and telemedicine as helpful compared to the base group aged 18–30. Conversely, LC patients older than 60 were more likely to perceive their professional environment as understanding, compared to the younger age group. Lastly, individuals with complementary private health insurance and a good economic situation were more likely to perceive their specialists and GPs encouraging their perception of the illness as helpful. Detailed results are presented in Tables 7 and SA4.

**Table 7:** Regression results for individual facilitators for adult long Covid‐19 patients in Austria.

	*Facilitators (I/II)*						
	My social environment and family were a great support to me	My professional environment was very understanding	The Long Covid Austria Facebook group provided useful information	The exchange with other patients helped me	My GP coordinated my treatment	GPs encouraged me in the perception of my illness	Specialists encouraged me in the perception of my illness
*Independent variables*	*Odds ratio [95% CI]*	*Odds ratio [95% CI]*	*Odds ratio [95% CI]*	*Odds ratio [95% CI]*	*Odds ratio [95% CI]*	*Odds ratio [95% CI]*	*Odds ratio [95% CI]*
Female gender	1.185 [0.661, 2.127]	1.013 [0.584, 1.756]	0.919 [0.466, 1.813]	1.094 [0.597, 2.007]	1.152 [0.575, 2.312]	1.146 [0.624, 2.102]	0.644 [0.367, 1.131]
*Aged 18–30 (base case)*							
Aged 31–40	0.98 [0.466, 2.062]	1.425 [0.732, 2.774]	0.728 [0.333, 1.591]	1.545 [0.782, 3.055]	0.522 [0.237, 1.15]	0.699 [0.348, 1.403]	1.245 [0.645, 2.403]
Aged 41–50	0.537 [0.267, 1.082]	1.569 [0.823, 2.991]	1.134 [0.524, 2.455]	1.296 [0.668, 2.517]	0.656 [0.313, 1.376]	0.749 [0.394, 1.427]	1.197 [0.638, 2.247]
Aged 51–60	0.447* [0.213, 0.937]	1.591 [0.791, 3.202]	1.085 [0.47, 2.503]	1.474 [0.714, 3.040]	0.604 [0.267, 1.365]	0.901 [0.445, 1.825]	1.116 [0.565, 2.205]
Older than 60	1.477 [0.338, 6.443]	6.567** [1.783, 24.186]	0.789 [0.161, 3.877]	2.954 [0.748, 11.669]	3.2 [0.868, 11.794]	1.522 [0.478, 4.850]	1.784 [0.507, 6.28]
Living in a rural area	1.154 [0.729, 1.825]	0.875 [0.568, 1.347]	0.917 [0.549, 1.533]	0.822 [0.518, 1.304]	1.061 [0.608, 1.853]	1.045 [0.653, 1.674]	0.791 [0.506, 1.237]
Health‐related background	1.053 [0.655, 1.693]	0.901 [0.576, 1.409]	1.012 [0.606, 1.69]	0.781 [0.489, 1.248]	1.371 [0.785, 2.394]	1.072 [0.662, 1.735]	0.704 [0.451, 1.100]
Complementary health insurance	1.085 [0.656, 1.796]	1.155 [0.718, 1.857]	0.945 [0.537, 1.664]	0.842 [0.509, 1.393]	1.137 [0.618, 2.091]	1.075 [0.647, 1.787]	1.859* [1.149, 3.007]
The household economic situation is good or very good	0.978 [0.518, 1.844]	0.966 [0.551, 1.694]	0.573 [0.283, 1.159]	0.583 [0.318, 1.067]	1.401 [0.701, 2.797]	2.031* [1.108, 3.720]	1.074 [0.611, 1.888]
*Household economic situation medium (base case)*							
The household economic situation is bad or very bad	0.448** [0.274, 0.734]	0.794 [0.497, 1.269]	0.852 [0.498, 1.458]	0.948 [0.577, 1.557]	1.177 [0.649, 2.137]	1.507 [0.896, 2.535]	0.953 [0.602, 1.509]
*N*	383	347	283	357	280	314	355

	*Facilitators (II/II)*		
	Doctors offered to shorten waiting times by switching to a private practice	Doctors offered to shorten waiting times through private co‐payments	Telemedicine facilitated my treatment
*Independent variables*	*Odds ratio [95% CI]*	*Odds ratio [95% CI]*	*Odds ratio [95% CI]*
Female gender	0.509 [0.167, 1.549]	1.042 [0.182, 5.962]	1.642 [0.709, 3.801]
*Aged 18–30 (base case)*			
Aged 31–40	0.628 [0.173, 2.283]	3.319 [0.428, 25.714]	0.550 [0.210, 1.443]
Aged 41–50	0.475 [0.136, 1.665]	2.154 [0.235, 19.727]	0.521 [0.211, 1.286]
Aged 51–60	0.104*** [0.020, 0.541]	0.610 [0.068, 5.458]	0.322* [0.119, 0.869]
Older than 60	0.890 [0.084, 9.446]	3.064 [0.055, 169.579]	0.212 [0.035, 1.274]
Living in a rural area	1.238 [0.492, 3.113]	1.351 [0.340, 5.370]	1.405 [0.743, 2.654]
Health‐related background	0.819 [0.290, 2.318]	1.459 [0.305, 6.981]	0.677 [0.342, 1.339]
Complementary health insurance	1.622 [0.620, 4.246]	2.714 [0.635, 11.612]	1.391 [0.694, 2.787]
The household economic situation is good or very good	1.415 [0.417, 4.793]	6.574 [0.654, 66.08]	0.652 [0.272, 1.565]
*Household economic situation medium (base case)*			
The household economic situation is bad or very bad	1.858 [0.691, 4.993]	0.678 [0.142, 3.243]	0.830 [0.422, 1.631]
*N*	103	72	184

*Note:* Red numbers refer to adverse patient experiences, green to beneficial ones; *N* varies by facilitator as only applicable participants are included in the analysis. Control variables included: Living in a federal state with central co‐ordination services, education, migration background, relationship status, prior physical or mental health conditions, number of perceived severe long Covid‐19 (LC) symptoms, year of LC onset, and LC duration.

* *p* value < 0.05

** *p* value < 0.01

*** *p* value < 0.001.

Results of all conducted sensitivity analyses were in line with the main analysis presented above.

### 3.4 Unmet Healthcare Needs

Overall, 72.3% (*n* = 313) of the surveyed LC patients reported having unmet healthcare needs, with 56.1% (*n* = 243), 70.2% (*n* = 304) and 59.8% (*n* = 259) reporting unmet healthcare needs for GPs, specialists and hospitals, respectively. The most commonly reported reasons with regard to GP care included not knowing a good GP (21.2% of participants) and geographic distance (13.4%). For specialists, costs were the main reason (25.2%), followed by the inability to identify a suitable specialist (12.5%) and waiting times for an appointment (11.8%). Lastly, with regard to hospital care, the main reasons included not knowing a suitable hospital (23.1%), waiting times (12.5%) and distance (10.6%). Detailed descriptive results can be found in Tables 8 and SA5.

**Table 8:** Unmet healthcare needs by healthcare service provider and reason.

	Overall *n* (%)	GP *n* (%)	Specialist *n* (%)	Hospital *n* (%)
No unmet healthcare needs	120 (27.7%)	190 (43.9%)	129 (29.8%)	174 (40.2%)
Unmet healthcare needs for any reason	313 (72.3%)	243 (56.1%)	304 (70.2%)	259 (59.8%)
Financial reasons		32 (7.4%)	109 (25.2%)	10 (2.3%)
Long waiting times		22 (5.1%)	51 (11.8%)	54 (12.5%)
No time (due to professional or caring responsibilities)		2 (0.5%)	2 (0.5%)	2 (0.5%)
Distance		58 (13.4%)	73 (16.9%)	46 (10.6%)
Fear		8 (1.8%)	4 (0.9%)	19 (4.4%)
Desire to wait and see if the problem improves by itself		17 (3.9%)	10 (2.3%)	13 (3.0%)
No known suitable provider		92 (21.2%)	54 (12.5%)	100 (23.1%)
Other		12 (2.8%)	1 (0.2%)	15 (3.5%)
Total	433	433	433	433

Looking at the socio‐economic and demographic differences in unmet healthcare needs, we find patients living in rural areas to be statistically significantly more likely to experience unmet needs regarding LC overall, irrespective of the service providers (GP, specialist and hospital). This also holds true in the detailed analyses for GP and specialist unmet needs. Moreover, individuals older than 60 were significantly less likely to experience GP‐related unmet needs and individuals in a good economic situation were less likely to do so for specialist and hospital care. Lastly, a health‐related background was a disadvantage regarding hospital unmet needs. Detailed results are presented in Table 9.

**Table 9:** Regression results for individual unmet healthcare needs for adult long Covid‐19 patients in Austria.

	Unmet healthcare needs		
	GP	Specialist	Hospital
Independent variables	Odds ratio [95% CI]	Odds ratio [95% CI]	Odds ratio [95% CI]
Female gender	0.761 [0.411, 1.408]	0.733 [0.367, 1.462]	0.765 [0.404, 1.446]
Aged 18–30 (base case)			
Aged 31–40	0.571 [0.282, 1.158]	0.504 [0.222, 1.143]	0.527 [0.253, 1.097]
Aged 41–50	0.808 [0.406, 1.609]	0.723 [0.323, 1.614]	0.684 [0.334, 1.398]
Aged 51–60	0.751 [0.358, 1.575]	0.530 [0.225, 1.249]	0.651 [0.302, 1.403]
Older than 60	0.196* [0.043, 0.892]	0.257 [0.060, 1.104]	0.743 [0.184, 3.006]
Living in a rural area	1.708* [1.063, 2.747]	1.788* [1.038, 3.078]	1.460 [0.895, 2.381]
Health‐related background	0.938 [0.581, 1.516]	1.331 [0.768, 2.307]	1.677* [1.014, 2.771]
Complementary health insurance	0.634 [0.382, 1.053]	0.782 [0.446, 1.368]	0.837 [0.496, 1.414]
Household economic situation good or very good	0.611 [0.337, 1.107]	0.483* [0.260, 0.898]	0.518* [0.284, 0.947]
Household economic situation medium (base case)			
Household economic situation bad or very bad	1.264 [0.764, 2.090]	1.615 [0.894, 2.918]	1.193 [0.708, 2.010]
*N*	387	387	387

*Notes:* Red numbers refer to adverse patient experiences, green to beneficial ones. Control variables included: Living in a federal state with central co‐ordination services, education, migration background, relationship status, prior physical or mental health conditions, number of perceived severe long Covid‐19 (LC) symptoms, year of LC onset, and LC duration. **p value < 0.01, ***p value < 0.001

* *p* value < 0.05.

## 4 Discussion

This study provides a comprehensive quantitative assessment of socio‐economic and demographic inequalities in healthcare access for LC patients in Austria. As part of a sequential exploratory mixed‐methods research effort, it builds on the findings of a prior qualitative study [16] and investigates barriers and facilitators to healthcare access as well as unmet healthcare needs. We find the overall barrier burden to be higher for individuals living in rural areas, those who do not have complementary private health insurance and those who are in a disadvantaged economic situation. These inequalities also prevail when investigating the 47 barriers individually, particularly regarding the availability and affordability of required health and social care services. The associations of urbanicity, complementary insurance and economic situation with the barriers were found to be consistent in direction. However, age and health‐related background through training or employment (e.g., nurses and veterinarians) were found to be connected to a higher or lower burden, depending on the individual barrier. Overall facilitator support, by contrast, did not differ by socio‐economic or demographic factors. At the same time, individual facilitator‐level variables such as age, complementary insurance and economic situation were statistically significantly associated with certain facilitators. Ultimately, we find that those living in rural areas were more likely to incur unmet healthcare needs regarding GP and specialist care. Being in a good economic situation reduced the respective probabilities for unmet specialist and hospital care needs.

Comparing our results with the socio‐economic and demographic characteristics initially identified in the previous qualitative study [16], age, urbanicity, health‐related background, complementary private health insurance and economic situation were found to be statistically significantly associated with healthcare access barriers. Contrary to the preceding findings [16], gender was not associated with access to healthcare. Considering the clear indication of gender‐based stigma emerging in the qualitative study, one potential explanation could be that both men and women in the quantitative study encountered the same barriers, for example, not being taken seriously by healthcare professionals. While this may have been the case for different reasons, such a difference might not have shown up in the quantitative study, as the questions did not include specific gender‐relevant aspects.

In the international context, our finding is in line with the only other quantitative study on LC access barriers and related inequalities from the Netherlands [21], which also does not report any gender differences in overall barrier burden. At the same time, on an individual barrier level, Brus et al. find women to be more likely to have problems with feeling uncomfortable asking for help, costs, reimbursements and eligibility of treatments, while men were more likely to encounter issues with availability and expertise of support. Brus et al. [21] find lower education and younger age to be associated with fewer encountered barriers overall, which is also in contrast to this study. Our findings on urbanicity are in line with international evidence identifying insufficient service availability in rural areas [53, 54]. Similarly, in the Austrian context, the Organisation for Economic Co‐operation and Development (OECD) [55] highlights the far higher physician density in Vienna compared to the other federal states. Yet, satisfaction with the health system did not differ by urbanicity but rather was found to be higher among those better educated and men [56].

In terms of the financial factor highlighted in this study, income‐related inequalities in healthcare access are well‐known internationally, although they vary significantly between countries and health systems [55]. We found income inequalities particularly in GP‐related barriers, as well as specialist and hospital unmet healthcare needs. This result is in stark contrast to prior research on Austria, which finds no significant inequalities in the probability of seeing a GP, while pro‐rich inequalities prevail for specialist visits [57, 58]. Our study, however, also identified income inequalities regarding barriers involving GPs, such as not being taken seriously by the GP, the GP not taking enough time for the consultation, and a lack of telemedicine or home visit options and diagnosis and treatment. Moreover, individuals in a good economic situation were more likely to perceive their GP's encouragement as helpful. Income‐related inequalities in GP care are particularly relevant in the case of LC, as the Austrian patient pathway is centred around GPs conducting the initial assessment and coordinating further diagnostics and treatment [59, 60].

Our results contribute to the currently limited, yet growing body of research on inequalities in LC healthcare access and thereby complement non‐LC inequalities research. We, moreover, incorporate novel perspectives based on the underlying exploratory study [16], such as assessing the role of health‐related background. LC healthcare access by medical personnel has been qualitatively researched, identifying the corresponding advantages, such as medical knowledge and connections, but also the disadvantages, including not being taken seriously [61]. Our study reflects these findings as well as healthcare access barriers to physicians emphasised in non‐LC research and connected to the respective professional environment, such as cultural issues [62].

### 4.1 Limitations and Strengths

This study has several limitations. First, as we are the first to provide a comprehensive characterisation of the Austrian LC population based on a convenience sample, no inferences on the representativeness of the patient sample can be made, emphasising the need for a national LC register. Second, due to the convenience sampling approach via intermediaries such as patient support groups and physicians, we do not know the response rate to our survey. Our sample, consequently, could be subject to non‐response and/or selection bias. Individuals of lower socio‐economic status have previously been found to be less likely to participate in surveys [63], which could be reflected in the above‐average share of highly educated individuals in our sample (57% above A‐levels compared to 21% [64] in the overall Austrian population). Our results thus potentially underestimate the true extent of LC healthcare access obstruction. Furthermore, our survey was limited to individuals with sufficient German language skills, which could limit the response of individuals with a migration background. Moreover, our sample is predominantly female, which, however, also reflects a higher LC prevalence among women [2]. Third, we included both active and recovered, self‐identified LC patients, potentially leading to recall or recruiting bias. However, the LC definition by the NICE was provided to participants for guidance in the respective explanatory text [41]. Fourth, due to the cross‐sectional design of this research, no inferences about causality can be made. Ultimately, we had to strike a balance in the necessary level of detail in translating our previous qualitative findings into a survey, while aiming to minimise the participant burden. Consequently, we were unable to explore in detail why patients, for example, felt like they were not being taken seriously by physicians, which could explain the absence of any gender‐based inequalities in our findings.

The study has two main strengths that enabled us to research inequalities in LC healthcare access. First, we provide a comprehensive characterisation of healthcare access by LC patients in a universal healthcare system. We investigate not only encountered barriers but also facilitators, as well as the unmet healthcare needs. To the best of our knowledge, this is the first study to do so, providing unique insights by combining the process of accessing care with the ultimate outcome: unmet needs. This process‐wise approach is essential as simply assessing unmet needs or utilisation may overlook patients who have overcome barriers. However, overcoming barriers is important, as the effort required to do so may negatively impact recovery and is subject to the identified inequalities, which therefore should be considered in policy recommendations to improve outcomes. Second, we accommodate the novelty of LC as a condition and its associated care pathways by building on an exploratory qualitative study. This approach allowed us to design our survey bottom‐up and tailored to the realities of LC patients. In terms of patient involvement and engagement, patients helped shape the research question and were directly involved in the design of the survey. Consequently, we were able to assess inequalities connected to six identified socio‐economic and demographic characteristics along 47 barriers and 10 facilitators, for example, using health‐related background as a variable of interest rather than education as identified and discussed previously [16]. Future patient involvement and engagement, however, should be extended to include the discussion of the results with patients, for example, to further improve interpretation, relevance and impact of the findings.

### 4.2 Policy and Research Implications

Our results bear important policy and research implications to reduce prevailing inequalities in LC care. The availability of services in rural areas, particularly targeted LC services, should be improved. This could be complemented by telemedicine or home visit offerings to ensure sufficient specialist coverage, especially given the often reduced mobility of LC patients. In this context, it is important that teleconsultations are taken seriously by expert assessors and are adequately reflected in respective reports, for example, for pension funds. Mobile specialised LC clinics could be a promising approach for the start in this regard, as they are currently being tested in a German federal state [65, 66]. Moreover, the availability of appropriate services covered by statutory health insurance should be improved to address the income‐related inequalities highlighted in this study. This is particularly pressing regarding GP care, and future policy efforts in Austria should consider that central case management by GPs may not be the most optimal set‐up after all, especially not without improved information, training, support and specialist referral opportunities. The latter issue was also highlighted in a previous Austrian study on the LC experiences of GPs [67].

Reducing inequalities has the potential to contribute to containing healthcare spending amid increasing life expectancy and strained public finances [68] and is imperative in light of the equality principle outlined in the Austrian health targets [69]. Further research is needed on the role of gender‐based inequalities, while the establishment of a national LC register poses both policy and research implications. Additionally, the underlying mechanisms of the income‐related inequalities in GP access should be further investigated. Our findings may also be applicable to other chronic diseases characterised by heterogeneous and fluctuating symptoms.

## 5 Conclusions

This study identified substantial socio‐economic and demographic inequalities in LC healthcare access in Austria. Our findings particularly contribute to research as we identified income‐related inequalities, particularly regarding GP care, contrasting prior non‐LC research. This study, hence, provides a call to action regarding the currently central role of GPs in LC care and how to adequately support them. Moreover, the improvement of the availability of services in rural areas and of offerings covered by statutory health insurance in both rural and urban areas are the main policy implications.

## Author Contributions

**Susanne Mayer:** conceptualisation, resource acquisition, analysis and survey, supervision, input and feedback, final manuscript approval. **Agata Łaszewska:** conceptualisation, resource acquisition, analysis and survey, input and feedback, final manuscript approval. **Peter Gamillscheg‐Müllner:** analysis and survey, piloting, data analysis, writing – original draft, input and feedback, final manuscript approval. **Kathryn Hoffmann:** expert contacts, survey design advice, input and feedback, final manuscript approval. **Sophie Diexer:** data analysis support, input and feedback, final manuscript approval. **Judit Simon:** writing – original draft, supervision, input and feedback, final manuscript approval.

## Disclosure

The funding bodies had no role in the design, methods, data collection, analysis, data interpretation or preparation of the article.

## Ethics Statement

All procedures performed in studies involving human participants were in accordance with the ethical standards of the institutional and national research committee and with the 1964 Helsinki Declaration and its later amendments or comparable ethical standards. The study was approved by the Ethics Committee of the Medical University of Vienna (EK 1228/2023).

## Consent

All participants provided informed consent to participate.

## Conflicts of Interest

The authors declare no conflicts of interest.

## Supporting information

ESM 1.

ESM 2.

## Data Availability

The dataset is available from the corresponding author upon reasonable request.
