# Supplementary material for: Theoretically Universal, Practically Unequal: Socio‐Economic Inequalities in Healthcare Access for Long Covid‐19 Patients in Austria
Source: Health Expect. 2026 Jan 8;29(1):e70553. doi: 10.1111/hex.70553 (PMC12780864; doi:10.1111/hex.70553)
Supplement: Supplementary file 1 — ESM 1. [file HEX-29-e70553-s002.docx]

## **Electronic Supplementary Material 1**

## ***Survey***

## ***0. Teilnahmekriterien (Jegliche „Nein“-Antwort als Ausschlusskriterium)***

1. **Ich bestätige, dass ich 18 Jahre alt oder älter bin**

- Ja
- Nein *– leider können Sie in diesem Fall nicht teilnehmen, danke für Ihr Interesse!*

1. **Ich bestätige, dass meine Deutschkenntnisse gut genug sind, um diesen Fragebogen zu beantworten**

- Ja
- Nein *– leider können Sie in diesem Fall nicht teilnehmen, danke für Ihr Interesse!*

1. **Ich bestätige, dass ich die Teilnehmer:innen-Information für diese Studie gelesen und verstanden habe**

- Ja
- Nein *– leider können Sie in diesem Fall nicht teilnehmen, danke für Ihr Interesse!*

1. **Sind oder waren Sie an long COVID-19 erkrankt?***Long COVID-19 bezeichnet anhaltende oder neu auftretende Symptome mindestens einen Monat nach einer COVID-19-Infektion. Diese können durch keine andere Diagnose erklärt werden und lagen vor der COVID-19-Infektion nicht vor.*

- Ja, ich bin seit ungefähr _________ *(Monat/Jahr)* an long COVID-19 erkrankt
- Ja, ich war von ungefähr __________ *(Monat/Jahr)* bis
  ungefähr _________ *(Monat/Jahr)* an long COVID-19 erkrankt und bin jetzt genesen
- Nein *– leider können Sie in diesem Fall nicht teilnehmen, danke für Ihr Interesse!*

## ***I. Allgemeine Informationen***

1. **Bitte geben Sie Ihr Geschlecht an:**

- Weiblich
- Männlich
- Divers
- Ich möchte diese Frage nicht beantworten

1. **Bitte geben Sie Ihr Alter an**
   ________ Jahre
2. **Bitte geben Sie das Bundesland an, in dem Sie leben**

- Burgenland
- Kärnten
- Niederösterreich
- Oberösterreich
- Salzburg
- Steiermark
- Tirol
- Vorarlberg
- Wien

1. **Wie würden Sie Ihren aktuellen Wohnort beschreiben?**

- Ländlich
- Städtisch

1. **Bitte geben Sie Ihre höchste abgeschlossene Ausbildung an**

- Pflichtschule
- Lehre
- Berufsbildende mittlere Schule oder Fachschule
- Matura
- Anderer Abschluss höher als Matura (z.B. Kolleg)
- Universität oder Fachhochschule
- Andere (*bitte angeben*): _____________________

1. **Haben Sie einen medizinischen Hintergrund?**

- Nein, ich habe keinerlei medizinischen Hintergrund
- Ja, ich habe einen medizinischen Hintergrund durch Ausbildung oder Beruf
  *(z.B. Ausbildung/Beruf als Sanitäter:in, Pflegeperson, Arzt/Ärztin, Ausbildung/Beruf in verwandten Feldern wie Veterinärmedizin oder Biologie, Tätigkeit im Medizinbereich mit anderer Ausbildung wie Verwaltung in Krankenhäusern oder Pharmaindustrie)*

1. **Haben anhaltende Symptome nach Ihrer COVID-19-Infektion zu einer Veränderung Ihrer beruflichen Situation und Ihrem Arbeitspensum geführt?**

- Nein, ich habe vorher nicht in einem Beruf gearbeitet (z. B. im Ruhestand, in Ausbildung, arbeitslos, Betreuungspflichten)
- Nein, ich arbeite genauso viele Stunden wie vorher
- Nein, aber ich bin derzeit im Krankenstand
- Ja, ich musste meine Arbeitszeit reduzieren
- Ja, ich musste meinen Job wechseln
- Ja, ich habe meinen Arbeitsplatz verloren und bin derzeit arbeitslos
- Anderes (*bitte ausführen*): ______________________

1. **Bitte um eine Selbsteinschätzung betreffend die Zeit *vor* Ihrer long COVID-19-Erkrankung: Wie sehen Sie die wirtschaftliche Lage Ihres Haushalts?**

- Sehr gut
- Gut
- Mittelmäßig
- Schlecht
- Sehr schlecht
- Ich möchte keine Angabe machen

1. **Bitte um eine Selbsteinschätzung betreffend die Zeit *während* Ihrer long COVID-19-Erkrankung: Wie sehen Sie die wirtschaftliche Lage Ihres Haushalts?**

- Sehr gut
- Gut
- Mittelmäßig
- Schlecht
- Sehr schlecht
- Ich möchte keine Angabe machen

1. **Haben Sie eine private Krankenzusatzversicherung?**

- Ja
- Nein
- Ich möchte keine Angabe machen

1. **Haben Sie Migrationshintergrund?***Bitte geben Sie an, falls Sie selbst oder mindestens ein Elternteil nicht in Österreich geboren wurde*

- Ja
- Nein
- Ich möchte keine Angabe machen

1. **Was ist ihr Familienstand?**

- Alleinstehend (z.B. ledig, verwitwet, geschieden)
- In einer Beziehung, verheiratet oder in eingetragener Partnerschaft, zusammenlebend
- In einer Beziehung, verheiratet oder in eingetragener Partnerschaft, getrennt lebend

1. **Haben Sie regelmäßige Betreuungspflichten?**

- Ja, mein Kind/meine Kinder
- Ja, sonstige Angehörige/nahestehende Personen
- Nein

1. **Hatten Sie vor Ihrer COVID-19 Infektion eine dauerhafte Krankheit oder ein chronisches Gesundheitsproblem?***Damit gemeint sind Krankheiten oder gesundheitliche Probleme, die mindestens sechs Monate andauern oder voraussichtlich andauern werden wie zum Beispiel Diabetes mellitus, Asthma oder Bluthochdruck*

- Ja, ich habe eine chronische Erkrankung
- Ja, ich habe mehrere chronische Erkrankungen
- Nein
- Ich möchte keine Angabe machen

1. **Waren Sie vor Ihrer COVID-19-Infektion wegen einer psychischen Erkrankung in medizinischer Behandlung oder haben Sie Unterstützung wegen einer psychischen Erkrankung erhalten?***Beispiele sind unter anderem Depressionen, Angststörungen, Demenz oder bipolare affektive Störungen*

- Ja
- Nein
- Ich möchte keine Angabe machen

## ***II. Long COVID-19 bezogene Fragen***

**Die nächste(n) Frage(n) beschäftigen sich mit Ihrem Alltag, allfälligen Einschränkungen und long COVID-19-Symptomen. Sie basieren auf der Forschung von Klok et al. (2020).**

1. **Können/konnten Sie alleine leben, ohne Unterstützung durch eine andere Person?
   *Bitte beziehen Sie sich auf den Zeitpunkt Ihrer long COVID Erkrankung, an dem Sie die schwerwiegendsten Symptome/Einschränkungen hatten.
   (z.B. selbständig essen, gehen, die Toilette benutzen und die tägliche Routinehygiene bewältigen können)***

- Ja
- Nein *– bitte überspringen Sie Fragen 21-23*

1. **Gibt/gab es Aufgaben/Aktivitäten zu Hause oder am Arbeitsplatz, die Sie nicht mehr selbst ausführen können/konnten?
   *Bitte beziehen Sie sich auf den Zeitpunkt Ihrer long COVID Erkrankung, an dem Sie die schwerwiegendsten Symptome/Einschränkungen hatten.***

- Ja *– bitte überspringen Sie Fragen 22-23*
- Nein

1. **Leiden/litten Sie unter Symptomen, Schmerzen, Depression oder Angstzuständen?
   *Bitte beziehen Sie sich auf den Zeitpunkt Ihrer long COVID Erkrankung, an dem Sie die schwerwiegendsten Symptome/Einschränkungen hatten.***

- Ja
- Nein *– bitte überspringen Sie Frage 23*

1. **Müssen/mussten Sie Aufgaben/Aktivitäten vermeiden, reduzieren oder über die Zeit verteilen?
   *Bitte beziehen Sie sich auf den Zeitpunkt Ihrer long COVID Erkrankung, an dem Sie die schwerwiegendsten Symptome/Einschränkungen hatten.***

- Ja
- Nein

1. **Bitte geben Sie an, welche Art von Symptomen Sie während Ihrer long COVID-19-Erkrankung in welchem Schweregrad erfahren haben (*ankreuzen*)**

| **Symptom** | **0 = keine Symptome** | **1** | **2** | **3** | **4** | **5 = maximaler Schweregrad** |
| --- | --- | --- | --- | --- | --- | --- |
| Anhaltende Erschöpfung |  |  |  |  |  |  |
| Eine Verschlechterung der Symptome nach körperlichen oder mentalen Aktivitäten (sog. "post exertional malaise/PEM") |  |  |  |  |  |  |
| Atembeschwerden |  |  |  |  |  |  |
| Muskel-/Gelenkbeschwerden |  |  |  |  |  |  |
| Verdauungsprobleme |  |  |  |  |  |  |
| Kognitive Beeinträchtigung |  |  |  |  |  |  |
| Kreislaufstörung/Herzrasen |  |  |  |  |  |  |
| Schlafstörung |  |  |  |  |  |  |
| Geruch- und Geschmacksstörung |  |  |  |  |  |  |
| Kopfschmerzen |  |  |  |  |  |  |
| Angst/Depression/Somatisierung |  |  |  |  |  |  |
| Andere Symptome: Bitte nennen |  |  |  |  |  |  |

## ***III. Fragen zu Ihren Erfahrungen mit dem Gesundheits- und Sozialsystem***

Die folgenden Fragen beschäftigen sich mit Zugangsbarrieren sowie unterstützenden Faktoren, die Ihnen die Inanspruchnahme von benötigten Gesundheits- und Sozialleistungen erschwert bzw. den Prozess dahin erleichtert haben. Bitte beziehen Sie sich hier ausschließlich auf Ihre Erfahrungen im Zuge Ihrer **long COVID-19-Erkrankung**!

1. Als Erstes möchten wir verstehen, ob und in welchem Ausmaß Sie Probleme bei der **Wahrnehmung Ihrer long COVID-19-Erkrankung** sowie **Informationen zur Krankheit und entsprechenden Behandlungsangeboten** hatten.

   Bitte geben Sie für jeden der folgenden Faktoren an, ob Sie hiermit keine Probleme, leichte Probleme oder große Probleme hatten *(ankreuzen)*.

   **Sollte ein Umstand auf Sie nicht zutreffen, wählen Sie bitte „Nicht zutreffend“ aus. Dies wäre beispielsweise der Fall, wenn sich eine Frage um Kinder dreht, Sie aber keine Kinder haben.**

|  | **Mögliche Barrieren** | Ich hatte damit **keine Probleme** | Ich hatte damit **leichte Probleme** | Ich hatte damit **große Probleme** | *Trifft auf mich nicht zu* |
| --- | --- | --- | --- | --- | --- |
| 1 | Zu wenig verfügbare Informationen betreffend die Krankheit und Behandlungsangebote |  |  |  |  |
| 2 | Schwierigkeiten, die Glaubwürdigkeit von Informationen einzuschätzen |  |  |  |  |
| 3 | Schwierigkeiten, Symptome als außergewöhnlich wahrzunehmen und der Krankheit zuzuordnen |  |  |  |  |

1. Als nächstes möchten wir verstehen, ob und in welchem Ausmaß Sie Probleme bei Ihrer **Suche nach Hilfe und entsprechenden Angeboten** zur Behandlung Ihrer long COVID-19-Erkrankung hatten.

   Bitte geben Sie für jeden der folgenden Faktoren an, ob Sie hiermit keine Probleme, leichte Probleme oder große Probleme hatten *(ankreuzen)*.

   **Sollte ein Umstand auf Sie nicht zutreffen, wählen Sie bitte „Nicht zutreffend“ aus. Dies wäre beispielsweise der Fall, wenn sich eine Frage um Kinder dreht, Sie allerdings keine Kinder haben.**

|  | **Mögliche Barrieren** | Ich hatte damit **keine Probleme** | Ich hatte damit **leichte Probleme** | Ich hatte damit **große Probleme** | *Trifft auf mich nicht zu* |
| --- | --- | --- | --- | --- | --- |
| 4 | Hausärzt:innen haben mich und meine Erkrankung nicht ernst genommen |  |  |  |  |
| 5 | Fachärzt:innen haben mich und meine Erkrankung nicht ernst genommen |  |  |  |  |
| 6 | Hausärzt:innen haben meine Symptome mit psychischen Problemen erklärt (z.B. Depressionen, psychosomatische Beschwerden) |  |  |  |  |
| 7 | Fachärzt:innen haben meine Symptome mit psychischen Problemen erklärt (z.B. Depressionen, psychosomatische Beschwerden) |  |  |  |  |
| 8 | Ärzt:innen oder Gutachter:innen haben Befunde von anderem ärztlichen Personal ignoriert oder nicht ernst genommen |  |  |  |  |
| 9 | Freunde oder Familie haben meine Erkrankung nicht ernst genommen |  |  |  |  |
| 10 | Mein berufliches Umfeld hat meine Erkrankung nicht ernst genommen |  |  |  |  |
| 11 | Ich habe mich selbst hinterfragt, ob ich wirklich krank bin |  |  |  |  |
| 12 | Ich kannte mich im Gesundheitssektor nicht gut aus und wusste nicht, wonach ich suchen soll |  |  |  |  |

1. Als nächstes möchten wir verstehen, ob und in welchem Ausmaß Sie Probleme mit dem **verfügbaren Angebot an Gesundheits- und Sozialleistungen** hatten.

   Bitte geben Sie für jeden der folgenden Faktoren an, ob Sie hiermit keine Probleme, leichte Probleme oder große Probleme hatten *(ankreuzen)*.

   **Sollte ein Umstand auf Sie nicht zutreffen, wählen Sie bitte „Nicht zutreffend“ aus. Dies wäre beispielsweise der Fall, wenn sich eine Frage um Kinder dreht, Sie allerdings keine Kinder haben.**

|  | **Mögliche Barrieren** | Ich hatte damit **keine Probleme** | Ich hatte damit **leichte Probleme** | Ich hatte damit **große Probleme** | *Trifft auf mich nicht zu* |
| --- | --- | --- | --- | --- | --- |
| 13 | Ambulanzen oder niedergelassene Fachärzt:innen erforderten eine Überweisung von anderen Ärzt:innen oder bestimmte Diagnosen |  |  |  |  |
| 14 | Um meiner Krankenkasse laufend meine Erkrankung mit Befunden nachzuweisen, musste ich öfter zu Ärzt:innen als notwendig |  |  |  |  |
| 15 | Hausärzt:innen akzeptierten keine neuen Patienten/Patientinnen oder hatten nur Termine in ferner Zukunft |  |  |  |  |
| 16 | Niedergelassene Fachärzt:innen akzeptierten keine neuen Patienten/Patientinnen oder hatten nur Termine in ferner Zukunft |  |  |  |  |
| 17 | Spitalsambulanzen inkl. long COVID-19-Ambulanzen akzeptierten keine neuen Patient:innen oder hatten nur Termine in ferner Zukunft |  |  |  |  |
| 18 | Hausärzt:innen haben sich zu wenig Zeit für mich genommen |  |  |  |  |
| 19 | Fachärzt:innen haben sich zu wenig Zeit für mich genommen |  |  |  |  |
| 20 | In der Spitalsambulanz wurde sich zu wenig Zeit für mich genommen |  |  |  |  |
| 21 | Long COVID-19 Ambulanzen, in denen ich vorstellig oder auf der Warteliste war, wurden geschlossen bevor meine Behandlung abgeschlossen war/begonnen hatte |  |  |  |  |
| 22 | Die Ausstattung der Wartebereiche bei Ärzt:innen bei denen ich war entsprach nicht meinen Bedürfnissen (z.B. zu laut, zu hell) |  |  |  |  |
| 23 | Die Anreise zu meinem Hausarzt/Hausärztin war eine Belastung (z.B. weit entfernt, öffentlich schlecht angebunden) |  |  |  |  |
|  |  |  |  |  |  |
|  | **Mögliche Barrieren** | Ich hatte damit **keine Probleme** | Ich hatte damit **leichte Probleme** | Ich hatte damit **große Probleme** | *Trifft auf mich nicht zu* |
| 24 | Die Anreise zu relevanten Fachärzt:innen war eine Belastung (z.B. weit entfernt, öffentlich schlecht angebunden) |  |  |  |  |
| 25 | Die Anreise zu relevanten Spitalsambulanzen war eine Belastung (z.B. weit entfernt, öffentlich schlecht angebunden) |  |  |  |  |
| 26 | Telemedizin oder Hausbesuche wurden von Hausärzt:innen nicht angeboten, obwohl ich es benötigt hätte |  |  |  |  |
| 27 | Telemedizin oder Hausbesuche wurden von Fachärzt:innen nicht angeboten, obwohl ich es benötigt hätte |  |  |  |  |
| 28 | Die Selbstorganisation meiner Behandlungen oder Amtswege war eine Belastung (z.B. Recherche von Angeboten, Terminvereinbarungen, Anträge) |  |  |  |  |

1. Als nächstes möchten wir verstehen, ob und in welchem Ausmaß Sie Probleme mit den **Kosten von benötigten Gesundheits- und Sozialleistungen** hatten. Hier beziehen wir uns auf die von Ihnen getragenen Kosten, die von keiner Versicherung/Krankenkasse übernommen wurden. Wir fragen Sie nicht nach konkreten Zahlen!

   Bitte geben Sie für jeden der folgenden Faktoren an, ob Sie hiermit keine Probleme, leichte Probleme oder große Probleme hatten *(ankreuzen)*.

   **Sollte ein Umstand auf Sie nicht zutreffen, wählen Sie bitte „Nicht zutreffend“ aus. Dies wäre beispielsweise der Fall, wenn sich eine Frage um Kinder dreht, Sie allerdings keine Kinder haben.**

|  | **Mögliche Barrieren** | Ich hatte damit **keine Probleme** | Ich hatte damit **leichte Probleme** | Ich hatte damit **große Probleme** | *Trifft auf mich nicht zu* |
| --- | --- | --- | --- | --- | --- |
| 29 | Die Kosten bei Hausärzt:innen waren eine finanzielle Belastung für mich |  |  |  |  |
| 30 | Die Kosten bei Fachärzt:innen waren eine finanzielle Belastung für mich |  |  |  |  |
| 31 | Die Kosten für Medikamente waren eine finanzielle Belastung für mich |  |  |  |  |
| 32 | Die Kosten für Nahrungsergänzungsmittel waren eine finanzielle Belastung für mich |  |  |  |  |
| 33 | Die Kosten für andere Behandlungen (z.B. Physiotherapie, Psychotherapie, Homöopathie) waren eine finanzielle Belastung für mich |  |  |  |  |
| 34 | Ich habe aufgrund der Kosten verschiedene Behandlungen gegeneinander abwägen müssen |  |  |  |  |
| 35 | Ich musste zu Wahl- oder Privatärzt:innen gehen, da ich keine Kassenärzt:innen finden konnte, die sich mit long COVID-19 auskannten |  |  |  |  |
| 36 | Ich musste aufgrund von Wartezeiten bei Kassenärzt:innen zu Wahl- oder Privatärzt:innen gehen |  |  |  |  |

1. Zuletzt möchten wir verstehen, ob und in welchem Ausmaß Sie Probleme mit der **Angemessenheit** **der genutzten Gesundheits- und Sozialleistungen** hatten.

   Bitte geben Sie für jeden der folgenden Faktoren an, ob Sie hiermit keine Probleme, leichte Probleme oder große Probleme hatten *(ankreuzen)*.

   **Sollte ein Umstand auf Sie nicht zutreffen, wählen Sie bitte „Nicht zutreffend“ aus. Dies wäre beispielsweise der Fall, wenn sich eine Frage um Kinder dreht, Sie allerdings keine Kinder haben.**

|  | **Mögliche Barrieren** | Ich hatte damit **keine Probleme** | Ich hatte damit **leichte Probleme** | Ich hatte damit **große Probleme** | *Trifft auf mich nicht zu* |
| --- | --- | --- | --- | --- | --- |
| 37 | Mein:e Hausarzt/Hausärztin hatte Schwierigkeiten, long COVID-19 zu diagnostizieren |  |  |  |  |
| 38 | Die Fachärzt:innen, bei denen ich war, hatten Schwierigkeiten, long COVID-19 zu diagnostizieren |  |  |  |  |
| 39 | Es gibt keinen diagnostischen Test/Biomarker über z.B. ein Blutbild, der eine eindeutige, objektive long COVID Diagnose ermöglicht |  |  |  |  |
| 40 | Mein:e Hausarzt/Hausärztin hatte Schwierigkeiten, meine Symptome zu behandeln |  |  |  |  |
| 41 | Die Fachärzt:innen, bei denen ich war, hatten Schwierigkeiten, meine Symptome zu behandeln |  |  |  |  |
| 42 | Ich hatte Schwierigkeiten, Krankengeld zu erhalten oder verlängern |  |  |  |  |
| 43 | Ich hatte Schwierigkeiten eine staatliche Berufsunfähigkeitspension zu erhalten |  |  |  |  |
| 44 | Ich hatte Schwierigkeiten eine private Berufsunfähigkeitspension zu erhalten |  |  |  |  |
| 45 | Ich hatte Schwierigkeiten, in Wiedereingliederungsteilzeit arbeiten zu können |  |  |  |  |
| 46 | Freunde und Familie hatten Schwierigkeiten, sich an meine long COVID-19-Einschränkungen anzupassen |  |  |  |  |
| 47 | Mein berufliches Umfeld hatte Schwierigkeiten, sich an meine long COVID-19-Einschränkungen anzupassen |  |  |  |  |

1. **Gibt es noch andere Barrieren, die Sie mit uns teilen möchten?**

____________________________________________________

1. **Kam es jemals vor, dass Sie medizinische Untersuchung(en) oder Behandlung(en), die Sie aufgrund Ihrer long COVID-19-Erkrankung benötigten, nicht in Anspruch nehmen konnten?**
   *Bitte denken Sie an alle medizinischen Untersuchungen oder Behandlungen ausgenommen zahnmedizinische Untersuchungen oder Behandlungen. Gründe können z.B. aber nicht nur Wartezeiten, mangelnde Angebote in der Nähe, oder zu hohe Kosten sein.*

- Ja
- Nein – *bitte überspringen Sie Frage 32*

1. **Bitte wählen Sie den für Sie jeweils *wichtigsten* Grund, warum Sie diese Untersuchung(en) oder Behandlung(en) bei i) Hausärzt:innen, ii) Niedergelassenen Fachärzt:innen, iii) in Krankenhäusern nicht** **in Anspruch nehmen konnten***Bitte wählen Sie pro Spalte entweder den wichtigsten Grund oder geben Sie an, dass Sie hier alle benötigten Untersuchungen oder Behandlungen in Anspruch nehmen konnten (ankreuzen)*

|  |  | Hausärzt:innen | Niedergelassene Fachärzt:innen | Krankenhäuser |
| --- | --- | --- | --- | --- |
| 1 | Finanzielle Gründe |  |  |  |
| 2 | Zu lange Wartezeiten |  |  |  |
| 3 | Hatte keine Zeit (berufliche Verpflichtungen oder Betreuungspflichten) |  |  |  |
| 4 | Erreichbarkeit nicht gegeben |  |  |  |
| 5 | Hatte zu große Angst |  |  |  |
| 6 | Wollte warten, ob das Problem von selbst besser wird |  |  |  |
| 7 | Kein guter Arzt/Ärztin/Krankenhaus bekannt |  |  |  |
| 8 | Anderer Grund |  |  |  |
| 9 | Ich konnte alle benötigten Untersuchungen oder Behandlungen in Anspruch nehmen |  |  |  |

1. Nun möchten wir in aller Kürze im Kontrast verstehen, ob und in welchem Ausmaß gewisse Faktoren Ihnen bei der **Inanspruchnahme von Gesundheits- und Sozialleistungen** **sowie einer Verbesserung Ihres Zustandes/Genesung** geholfen haben.

   Bitte geben Sie für jeden der folgenden Faktoren an, ob diese für Sie keine Hilfe war, Ihnen ein bisschen geholfen hat oder Ihnen sehr geholfen hat *(ankreuzen)*.

   **Sollte ein Umstand auf Sie nicht zutreffen, wählen Sie bitte „Nicht zutreffend“ aus. Dies wäre beispielsweise der Fall, wenn sich eine Frage um Kinder dreht, Sie allerdings keine Kinder haben.**

|  | **Unterstützende Faktoren** | Das war **keine Hilfe** für mich | Das hat mir **ein bisschen** geholfen | Das hat mir **sehr** geholfen | *Trifft auf mich nicht zu* |
| --- | --- | --- | --- | --- | --- |
| 1 | Mein soziales Umfeld und Familie waren mir eine große Stütze |  |  |  |  |
| 2 | Mein professionelles Umfeld war sehr verständnisvoll |  |  |  |  |
| 3 | Die *Long Covid Austria* Facebook Gruppe hat nützliche Informationen bereitgestellt |  |  |  |  |
| 4 | Der Austausch mit anderen Patient:innen hat mir geholfen |  |  |  |  |
| 5 | Mein:e Hausarzt/Hausärztin hat meine Behandlung koordiniert |  |  |  |  |
| 6 | Telemedizin hat meine Behandlung erleichtert |  |  |  |  |
| 7 | Hausärzt:innen haben mich in der Wahrnehmung meiner Erkrankung bestärkt |  |  |  |  |
| 8 | Fachärzt:innen haben mich in der Wahrnehmung meiner Erkrankung bestärkt |  |  |  |  |
| 9 | Ärzt:innen haben mir angeboten, Wartezeiten durch einen Wechsel in die Privatordination zu verkürzen |  |  |  |  |
| 10 | Ärzt:innen haben mir angeboten, Wartezeiten durch private Zuzahlungen zu verkürzen |  |  |  |  |

1. **Gibt es noch andere unterstützende Faktoren, die Sie mit uns teilen möchten?**

_________________________________________________________________

## ***0. Teilnahmekriterien (Jegliche „Nein“-Antwort als Ausschlusskriterium)***

1. **Ich bestätige, dass ich 18 Jahre alt oder älter bin**

- Ja
- Nein *– leider können Sie in diesem Fall nicht teilnehmen, danke für Ihr Interesse!*

1. **Ich bestätige, dass meine Deutschkenntnisse gut genug sind, um diesen Fragebogen zu beantworten**

- Ja
- Nein *– leider können Sie in diesem Fall nicht teilnehmen, danke für Ihr Interesse!*

1. **Ich bestätige, dass ich die Teilnehmer:innen-Information für diese Studie gelesen und verstanden habe**

- Ja
- Nein *– leider können Sie in diesem Fall nicht teilnehmen, danke für Ihr Interesse!*

1. **Sind oder waren Sie an long COVID-19 erkrankt?***Long COVID-19 bezeichnet anhaltende oder neu auftretende Symptome mindestens einen Monat nach einer COVID-19-Infektion. Diese können durch keine andere Diagnose erklärt werden und lagen vor der COVID-19-Infektion nicht vor.*

- Ja, ich bin seit ungefähr _________ *(Monat/Jahr)* an long COVID-19 erkrankt
- Ja, ich war von ungefähr __________ *(Monat/Jahr)* bis
  ungefähr _________ *(Monat/Jahr)* an long COVID-19 erkrankt und bin jetzt genesen
- Nein *– leider können Sie in diesem Fall nicht teilnehmen, danke für Ihr Interesse!*

## ***I. Allgemeine Informationen***

1. **Bitte geben Sie Ihr Geschlecht an:**

- Weiblich
- Männlich
- Divers
- Ich möchte diese Frage nicht beantworten

1. **Bitte geben Sie Ihr Alter an**
   ________ Jahre
2. **Bitte geben Sie das Bundesland an, in dem Sie leben**

- Burgenland
- Kärnten
- Niederösterreich
- Oberösterreich
- Salzburg
- Steiermark
- Tirol
- Vorarlberg
- Wien

1. **Wie würden Sie Ihren aktuellen Wohnort beschreiben?**

- Ländlich
- Städtisch

1. **Bitte geben Sie Ihre höchste abgeschlossene Ausbildung an**

- Pflichtschule
- Lehre
- Berufsbildende mittlere Schule oder Fachschule
- Matura
- Anderer Abschluss höher als Matura (z.B. Kolleg)
- Universität oder Fachhochschule
- Andere (*bitte angeben*): _____________________

1. **Haben Sie einen medizinischen Hintergrund?**

- Nein, ich habe keinerlei medizinischen Hintergrund
- Ja, ich habe einen medizinischen Hintergrund durch Ausbildung oder Beruf
  *(z.B. Ausbildung/Beruf als Sanitäter:in, Pflegeperson, Arzt/Ärztin, Ausbildung/Beruf in verwandten Feldern wie Veterinärmedizin oder Biologie, Tätigkeit im Medizinbereich mit anderer Ausbildung wie Verwaltung in Krankenhäusern oder Pharmaindustrie)*

1. **Haben anhaltende Symptome nach Ihrer COVID-19-Infektion zu einer Veränderung Ihrer beruflichen Situation und Ihrem Arbeitspensum geführt?**

- Nein, ich habe vorher nicht in einem Beruf gearbeitet (z. B. im Ruhestand, in Ausbildung, arbeitslos, Betreuungspflichten)
- Nein, ich arbeite genauso viele Stunden wie vorher
- Nein, aber ich bin derzeit im Krankenstand
- Ja, ich musste meine Arbeitszeit reduzieren
- Ja, ich musste meinen Job wechseln
- Ja, ich habe meinen Arbeitsplatz verloren und bin derzeit arbeitslos
- Anderes (*bitte ausführen*): ______________________

1. **Bitte um eine Selbsteinschätzung betreffend die Zeit *vor* Ihrer long COVID-19-Erkrankung: Wie sehen Sie die wirtschaftliche Lage Ihres Haushalts?**

- Sehr gut
- Gut
- Mittelmäßig
- Schlecht
- Sehr schlecht
- Ich möchte keine Angabe machen

1. **Bitte um eine Selbsteinschätzung betreffend die Zeit *während* Ihrer long COVID-19-Erkrankung: Wie sehen Sie die wirtschaftliche Lage Ihres Haushalts?**

- Sehr gut
- Gut
- Mittelmäßig
- Schlecht
- Sehr schlecht
- Ich möchte keine Angabe machen

1. **Haben Sie eine private Krankenzusatzversicherung?**

- Ja
- Nein
- Ich möchte keine Angabe machen

1. **Haben Sie Migrationshintergrund?***Bitte geben Sie an, falls Sie selbst oder mindestens ein Elternteil nicht in Österreich geboren wurde*

- Ja
- Nein
- Ich möchte keine Angabe machen

1. **Was ist ihr Familienstand?**

- Alleinstehend (z.B. ledig, verwitwet, geschieden)
- In einer Beziehung, verheiratet oder in eingetragener Partnerschaft, zusammenlebend
- In einer Beziehung, verheiratet oder in eingetragener Partnerschaft, getrennt lebend

1. **Haben Sie regelmäßige Betreuungspflichten?**

- Ja, mein Kind/meine Kinder
- Ja, sonstige Angehörige/nahestehende Personen
- Nein

1. **Hatten Sie vor Ihrer COVID-19 Infektion eine dauerhafte Krankheit oder ein chronisches Gesundheitsproblem?***Damit gemeint sind Krankheiten oder gesundheitliche Probleme, die mindestens sechs Monate andauern oder voraussichtlich andauern werden wie zum Beispiel Diabetes mellitus, Asthma oder Bluthochdruck*

- Ja, ich habe eine chronische Erkrankung
- Ja, ich habe mehrere chronische Erkrankungen
- Nein
- Ich möchte keine Angabe machen

1. **Waren Sie vor Ihrer COVID-19-Infektion wegen einer psychischen Erkrankung in medizinischer Behandlung oder haben Sie Unterstützung wegen einer psychischen Erkrankung erhalten?***Beispiele sind unter anderem Depressionen, Angststörungen, Demenz oder bipolare affektive Störungen*

- Ja
- Nein
- Ich möchte keine Angabe machen

## ***II. Long COVID-19 bezogene Fragen***

**Die nächste(n) Frage(n) beschäftigen sich mit Ihrem Alltag, allfälligen Einschränkungen und long COVID-19-Symptomen. Sie basieren auf der Forschung von Klok et al. (2020).**

1. **Können/konnten Sie alleine leben, ohne Unterstützung durch eine andere Person?
   *Bitte beziehen Sie sich auf den Zeitpunkt Ihrer long COVID Erkrankung, an dem Sie die schwerwiegendsten Symptome/Einschränkungen hatten.
   (z.B. selbständig essen, gehen, die Toilette benutzen und die tägliche Routinehygiene bewältigen können)***

- Ja
- Nein *– bitte überspringen Sie Fragen 21-23*

1. **Gibt/gab es Aufgaben/Aktivitäten zu Hause oder am Arbeitsplatz, die Sie nicht mehr selbst ausführen können/konnten?
   *Bitte beziehen Sie sich auf den Zeitpunkt Ihrer long COVID Erkrankung, an dem Sie die schwerwiegendsten Symptome/Einschränkungen hatten.***

- Ja *– bitte überspringen Sie Fragen 22-23*
- Nein

1. **Leiden/litten Sie unter Symptomen, Schmerzen, Depression oder Angstzuständen?
   *Bitte beziehen Sie sich auf den Zeitpunkt Ihrer long COVID Erkrankung, an dem Sie die schwerwiegendsten Symptome/Einschränkungen hatten.***

- Ja
- Nein *– bitte überspringen Sie Frage 23*

1. **Müssen/mussten Sie Aufgaben/Aktivitäten vermeiden, reduzieren oder über die Zeit verteilen?
   *Bitte beziehen Sie sich auf den Zeitpunkt Ihrer long COVID Erkrankung, an dem Sie die schwerwiegendsten Symptome/Einschränkungen hatten.***

- Ja
- Nein

1. **Bitte geben Sie an, welche Art von Symptomen Sie während Ihrer long COVID-19-Erkrankung in welchem Schweregrad erfahren haben (*ankreuzen*)**

| **Symptom** | **0 = keine Symptome** | **1** | **2** | **3** | **4** | **5 = maximaler Schweregrad** |
| --- | --- | --- | --- | --- | --- | --- |
| Anhaltende Erschöpfung |  |  |  |  |  |  |
| Eine Verschlechterung der Symptome nach körperlichen oder mentalen Aktivitäten (sog. "post exertional malaise/PEM") |  |  |  |  |  |  |
| Atembeschwerden |  |  |  |  |  |  |
| Muskel-/Gelenkbeschwerden |  |  |  |  |  |  |
| Verdauungsprobleme |  |  |  |  |  |  |
| Kognitive Beeinträchtigung |  |  |  |  |  |  |
| Kreislaufstörung/Herzrasen |  |  |  |  |  |  |
| Schlafstörung |  |  |  |  |  |  |
| Geruch- und Geschmacksstörung |  |  |  |  |  |  |
| Kopfschmerzen |  |  |  |  |  |  |
| Angst/Depression/Somatisierung |  |  |  |  |  |  |
| Andere Symptome: Bitte nennen |  |  |  |  |  |  |

## ***III. Fragen zu Ihren Erfahrungen mit dem Gesundheits- und Sozialsystem***

Die folgenden Fragen beschäftigen sich mit Zugangsbarrieren sowie unterstützenden Faktoren, die Ihnen die Inanspruchnahme von benötigten Gesundheits- und Sozialleistungen erschwert bzw. den Prozess dahin erleichtert haben. Bitte beziehen Sie sich hier ausschließlich auf Ihre Erfahrungen im Zuge Ihrer **long COVID-19-Erkrankung**!

1. Als Erstes möchten wir verstehen, ob und in welchem Ausmaß Sie Probleme bei der **Wahrnehmung Ihrer long COVID-19-Erkrankung** sowie **Informationen zur Krankheit und entsprechenden Behandlungsangeboten** hatten.

   Bitte geben Sie für jeden der folgenden Faktoren an, ob Sie hiermit keine Probleme, leichte Probleme oder große Probleme hatten *(ankreuzen)*.

   **Sollte ein Umstand auf Sie nicht zutreffen, wählen Sie bitte „Nicht zutreffend“ aus. Dies wäre beispielsweise der Fall, wenn sich eine Frage um Kinder dreht, Sie aber keine Kinder haben.**

|  | **Mögliche Barrieren** | Ich hatte damit **keine Probleme** | Ich hatte damit **leichte Probleme** | Ich hatte damit **große Probleme** | *Trifft auf mich nicht zu* |
| --- | --- | --- | --- | --- | --- |
| 1 | Zu wenig verfügbare Informationen betreffend die Krankheit und Behandlungsangebote |  |  |  |  |
| 2 | Schwierigkeiten, die Glaubwürdigkeit von Informationen einzuschätzen |  |  |  |  |
| 3 | Schwierigkeiten, Symptome als außergewöhnlich wahrzunehmen und der Krankheit zuzuordnen |  |  |  |  |

1. Als nächstes möchten wir verstehen, ob und in welchem Ausmaß Sie Probleme bei Ihrer **Suche nach Hilfe und entsprechenden Angeboten** zur Behandlung Ihrer long COVID-19-Erkrankung hatten.

   Bitte geben Sie für jeden der folgenden Faktoren an, ob Sie hiermit keine Probleme, leichte Probleme oder große Probleme hatten *(ankreuzen)*.

   **Sollte ein Umstand auf Sie nicht zutreffen, wählen Sie bitte „Nicht zutreffend“ aus. Dies wäre beispielsweise der Fall, wenn sich eine Frage um Kinder dreht, Sie allerdings keine Kinder haben.**

|  | **Mögliche Barrieren** | Ich hatte damit **keine Probleme** | Ich hatte damit **leichte Probleme** | Ich hatte damit **große Probleme** | *Trifft auf mich nicht zu* |
| --- | --- | --- | --- | --- | --- |
| 4 | Hausärzt:innen haben mich und meine Erkrankung nicht ernst genommen |  |  |  |  |
| 5 | Fachärzt:innen haben mich und meine Erkrankung nicht ernst genommen |  |  |  |  |
| 6 | Hausärzt:innen haben meine Symptome mit psychischen Problemen erklärt (z.B. Depressionen, psychosomatische Beschwerden) |  |  |  |  |
| 7 | Fachärzt:innen haben meine Symptome mit psychischen Problemen erklärt (z.B. Depressionen, psychosomatische Beschwerden) |  |  |  |  |
| 8 | Ärzt:innen oder Gutachter:innen haben Befunde von anderem ärztlichen Personal ignoriert oder nicht ernst genommen |  |  |  |  |
| 9 | Freunde oder Familie haben meine Erkrankung nicht ernst genommen |  |  |  |  |
| 10 | Mein berufliches Umfeld hat meine Erkrankung nicht ernst genommen |  |  |  |  |
| 11 | Ich habe mich selbst hinterfragt, ob ich wirklich krank bin |  |  |  |  |
| 12 | Ich kannte mich im Gesundheitssektor nicht gut aus und wusste nicht, wonach ich suchen soll |  |  |  |  |

1. Als nächstes möchten wir verstehen, ob und in welchem Ausmaß Sie Probleme mit dem **verfügbaren Angebot an Gesundheits- und Sozialleistungen** hatten.

   Bitte geben Sie für jeden der folgenden Faktoren an, ob Sie hiermit keine Probleme, leichte Probleme oder große Probleme hatten *(ankreuzen)*.

   **Sollte ein Umstand auf Sie nicht zutreffen, wählen Sie bitte „Nicht zutreffend“ aus. Dies wäre beispielsweise der Fall, wenn sich eine Frage um Kinder dreht, Sie allerdings keine Kinder haben.**

|  | **Mögliche Barrieren** | Ich hatte damit **keine Probleme** | Ich hatte damit **leichte Probleme** | Ich hatte damit **große Probleme** | *Trifft auf mich nicht zu* |
| --- | --- | --- | --- | --- | --- |
| 13 | Ambulanzen oder niedergelassene Fachärzt:innen erforderten eine Überweisung von anderen Ärzt:innen oder bestimmte Diagnosen |  |  |  |  |
| 14 | Um meiner Krankenkasse laufend meine Erkrankung mit Befunden nachzuweisen, musste ich öfter zu Ärzt:innen als notwendig |  |  |  |  |
| 15 | Hausärzt:innen akzeptierten keine neuen Patienten/Patientinnen oder hatten nur Termine in ferner Zukunft |  |  |  |  |
| 16 | Niedergelassene Fachärzt:innen akzeptierten keine neuen Patienten/Patientinnen oder hatten nur Termine in ferner Zukunft |  |  |  |  |
| 17 | Spitalsambulanzen inkl. long COVID-19-Ambulanzen akzeptierten keine neuen Patient:innen oder hatten nur Termine in ferner Zukunft |  |  |  |  |
| 18 | Hausärzt:innen haben sich zu wenig Zeit für mich genommen |  |  |  |  |
| 19 | Fachärzt:innen haben sich zu wenig Zeit für mich genommen |  |  |  |  |
| 20 | In der Spitalsambulanz wurde sich zu wenig Zeit für mich genommen |  |  |  |  |
| 21 | Long COVID-19 Ambulanzen, in denen ich vorstellig oder auf der Warteliste war, wurden geschlossen bevor meine Behandlung abgeschlossen war/begonnen hatte |  |  |  |  |
| 22 | Die Ausstattung der Wartebereiche bei Ärzt:innen bei denen ich war entsprach nicht meinen Bedürfnissen (z.B. zu laut, zu hell) |  |  |  |  |
| 23 | Die Anreise zu meinem Hausarzt/Hausärztin war eine Belastung (z.B. weit entfernt, öffentlich schlecht angebunden) |  |  |  |  |
|  |  |  |  |  |  |
|  | **Mögliche Barrieren** | Ich hatte damit **keine Probleme** | Ich hatte damit **leichte Probleme** | Ich hatte damit **große Probleme** | *Trifft auf mich nicht zu* |
| 24 | Die Anreise zu relevanten Fachärzt:innen war eine Belastung (z.B. weit entfernt, öffentlich schlecht angebunden) |  |  |  |  |
| 25 | Die Anreise zu relevanten Spitalsambulanzen war eine Belastung (z.B. weit entfernt, öffentlich schlecht angebunden) |  |  |  |  |
| 26 | Telemedizin oder Hausbesuche wurden von Hausärzt:innen nicht angeboten, obwohl ich es benötigt hätte |  |  |  |  |
| 27 | Telemedizin oder Hausbesuche wurden von Fachärzt:innen nicht angeboten, obwohl ich es benötigt hätte |  |  |  |  |
| 28 | Die Selbstorganisation meiner Behandlungen oder Amtswege war eine Belastung (z.B. Recherche von Angeboten, Terminvereinbarungen, Anträge) |  |  |  |  |

1. Als nächstes möchten wir verstehen, ob und in welchem Ausmaß Sie Probleme mit den **Kosten von benötigten Gesundheits- und Sozialleistungen** hatten. Hier beziehen wir uns auf die von Ihnen getragenen Kosten, die von keiner Versicherung/Krankenkasse übernommen wurden. Wir fragen Sie nicht nach konkreten Zahlen!

   Bitte geben Sie für jeden der folgenden Faktoren an, ob Sie hiermit keine Probleme, leichte Probleme oder große Probleme hatten *(ankreuzen)*.

   **Sollte ein Umstand auf Sie nicht zutreffen, wählen Sie bitte „Nicht zutreffend“ aus. Dies wäre beispielsweise der Fall, wenn sich eine Frage um Kinder dreht, Sie allerdings keine Kinder haben.**

|  | **Mögliche Barrieren** | Ich hatte damit **keine Probleme** | Ich hatte damit **leichte Probleme** | Ich hatte damit **große Probleme** | *Trifft auf mich nicht zu* |
| --- | --- | --- | --- | --- | --- |
| 29 | Die Kosten bei Hausärzt:innen waren eine finanzielle Belastung für mich |  |  |  |  |
| 30 | Die Kosten bei Fachärzt:innen waren eine finanzielle Belastung für mich |  |  |  |  |
| 31 | Die Kosten für Medikamente waren eine finanzielle Belastung für mich |  |  |  |  |
| 32 | Die Kosten für Nahrungsergänzungsmittel waren eine finanzielle Belastung für mich |  |  |  |  |
| 33 | Die Kosten für andere Behandlungen (z.B. Physiotherapie, Psychotherapie, Homöopathie) waren eine finanzielle Belastung für mich |  |  |  |  |
| 34 | Ich habe aufgrund der Kosten verschiedene Behandlungen gegeneinander abwägen müssen |  |  |  |  |
| 35 | Ich musste zu Wahl- oder Privatärzt:innen gehen, da ich keine Kassenärzt:innen finden konnte, die sich mit long COVID-19 auskannten |  |  |  |  |
| 36 | Ich musste aufgrund von Wartezeiten bei Kassenärzt:innen zu Wahl- oder Privatärzt:innen gehen |  |  |  |  |

1. Zuletzt möchten wir verstehen, ob und in welchem Ausmaß Sie Probleme mit der **Angemessenheit** **der genutzten Gesundheits- und Sozialleistungen** hatten.

   Bitte geben Sie für jeden der folgenden Faktoren an, ob Sie hiermit keine Probleme, leichte Probleme oder große Probleme hatten *(ankreuzen)*.

   **Sollte ein Umstand auf Sie nicht zutreffen, wählen Sie bitte „Nicht zutreffend“ aus. Dies wäre beispielsweise der Fall, wenn sich eine Frage um Kinder dreht, Sie allerdings keine Kinder haben.**

|  | **Mögliche Barrieren** | Ich hatte damit **keine Probleme** | Ich hatte damit **leichte Probleme** | Ich hatte damit **große Probleme** | *Trifft auf mich nicht zu* |
| --- | --- | --- | --- | --- | --- |
| 37 | Mein:e Hausarzt/Hausärztin hatte Schwierigkeiten, long COVID-19 zu diagnostizieren |  |  |  |  |
| 38 | Die Fachärzt:innen, bei denen ich war, hatten Schwierigkeiten, long COVID-19 zu diagnostizieren |  |  |  |  |
| 39 | Es gibt keinen diagnostischen Test/Biomarker über z.B. ein Blutbild, der eine eindeutige, objektive long COVID Diagnose ermöglicht |  |  |  |  |
| 40 | Mein:e Hausarzt/Hausärztin hatte Schwierigkeiten, meine Symptome zu behandeln |  |  |  |  |
| 41 | Die Fachärzt:innen, bei denen ich war, hatten Schwierigkeiten, meine Symptome zu behandeln |  |  |  |  |
| 42 | Ich hatte Schwierigkeiten, Krankengeld zu erhalten oder verlängern |  |  |  |  |
| 43 | Ich hatte Schwierigkeiten eine staatliche Berufsunfähigkeitspension zu erhalten |  |  |  |  |
| 44 | Ich hatte Schwierigkeiten eine private Berufsunfähigkeitspension zu erhalten |  |  |  |  |
| 45 | Ich hatte Schwierigkeiten, in Wiedereingliederungsteilzeit arbeiten zu können |  |  |  |  |
| 46 | Freunde und Familie hatten Schwierigkeiten, sich an meine long COVID-19-Einschränkungen anzupassen |  |  |  |  |
| 47 | Mein berufliches Umfeld hatte Schwierigkeiten, sich an meine long COVID-19-Einschränkungen anzupassen |  |  |  |  |

1. **Gibt es noch andere Barrieren, die Sie mit uns teilen möchten?**

____________________________________________________

1. **Kam es jemals vor, dass Sie medizinische Untersuchung(en) oder Behandlung(en), die Sie aufgrund Ihrer long COVID-19-Erkrankung benötigten, nicht in Anspruch nehmen konnten?**
   *Bitte denken Sie an alle medizinischen Untersuchungen oder Behandlungen ausgenommen zahnmedizinische Untersuchungen oder Behandlungen. Gründe können z.B. aber nicht nur Wartezeiten, mangelnde Angebote in der Nähe, oder zu hohe Kosten sein.*

- Ja
- Nein – *bitte überspringen Sie Frage 32*

1. **Bitte wählen Sie den für Sie jeweils *wichtigsten* Grund, warum Sie diese Untersuchung(en) oder Behandlung(en) bei i) Hausärzt:innen, ii) Niedergelassenen Fachärzt:innen, iii) in Krankenhäusern nicht** **in Anspruch nehmen konnten***Bitte wählen Sie pro Spalte entweder den wichtigsten Grund oder geben Sie an, dass Sie hier alle benötigten Untersuchungen oder Behandlungen in Anspruch nehmen konnten (ankreuzen)*

|  |  | Hausärzt:innen | Niedergelassene Fachärzt:innen | Krankenhäuser |
| --- | --- | --- | --- | --- |
| 1 | Finanzielle Gründe |  |  |  |
| 2 | Zu lange Wartezeiten |  |  |  |
| 3 | Hatte keine Zeit (berufliche Verpflichtungen oder Betreuungspflichten) |  |  |  |
| 4 | Erreichbarkeit nicht gegeben |  |  |  |
| 5 | Hatte zu große Angst |  |  |  |
| 6 | Wollte warten, ob das Problem von selbst besser wird |  |  |  |
| 7 | Kein guter Arzt/Ärztin/Krankenhaus bekannt |  |  |  |
| 8 | Anderer Grund |  |  |  |
| 9 | Ich konnte alle benötigten Untersuchungen oder Behandlungen in Anspruch nehmen |  |  |  |

1. Nun möchten wir in aller Kürze im Kontrast verstehen, ob und in welchem Ausmaß gewisse Faktoren Ihnen bei der **Inanspruchnahme von Gesundheits- und Sozialleistungen** **sowie einer Verbesserung Ihres Zustandes/Genesung** geholfen haben.

   Bitte geben Sie für jeden der folgenden Faktoren an, ob diese für Sie keine Hilfe war, Ihnen ein bisschen geholfen hat oder Ihnen sehr geholfen hat *(ankreuzen)*.

   **Sollte ein Umstand auf Sie nicht zutreffen, wählen Sie bitte „Nicht zutreffend“ aus. Dies wäre beispielsweise der Fall, wenn sich eine Frage um Kinder dreht, Sie allerdings keine Kinder haben.**

|  | **Unterstützende Faktoren** | Das war **keine Hilfe** für mich | Das hat mir **ein bisschen** geholfen | Das hat mir **sehr** geholfen | *Trifft auf mich nicht zu* |
| --- | --- | --- | --- | --- | --- |
| 1 | Mein soziales Umfeld und Familie waren mir eine große Stütze |  |  |  |  |
| 2 | Mein professionelles Umfeld war sehr verständnisvoll |  |  |  |  |
| 3 | Die *Long Covid Austria* Facebook Gruppe hat nützliche Informationen bereitgestellt |  |  |  |  |
| 4 | Der Austausch mit anderen Patient:innen hat mir geholfen |  |  |  |  |
| 5 | Mein:e Hausarzt/Hausärztin hat meine Behandlung koordiniert |  |  |  |  |
| 6 | Telemedizin hat meine Behandlung erleichtert |  |  |  |  |
| 7 | Hausärzt:innen haben mich in der Wahrnehmung meiner Erkrankung bestärkt |  |  |  |  |
| 8 | Fachärzt:innen haben mich in der Wahrnehmung meiner Erkrankung bestärkt |  |  |  |  |
| 9 | Ärzt:innen haben mir angeboten, Wartezeiten durch einen Wechsel in die Privatordination zu verkürzen |  |  |  |  |
| 10 | Ärzt:innen haben mir angeboten, Wartezeiten durch private Zuzahlungen zu verkürzen |  |  |  |  |

1. **Gibt es noch andere unterstützende Faktoren, die Sie mit uns teilen möchten?**

_________________________________________________________________
