# Supplementary material for: Theoretically Universal, Practically Unequal: Socio‐Economic Inequalities in Healthcare Access for Long Covid‐19 Patients in Austria
Source: Health Expect. 2026 Jan 8;29(1):e70553. doi: 10.1111/hex.70553 (PMC12780864; doi:10.1111/hex.70553)
Supplement: Supplementary file 2 — ESM 2. [file HEX-29-e70553-s001.docx]

## **Electronic Supplementary Material 2**

Table A1. STROBE Statement—Checklist of items that should be included in reports of cross-sectional studies

|  | Item No | Recommendation | Page No |
| --- | --- | --- | --- |
| **Title and abstract** | 1 | (*a*) Indicate the study’s design with a commonly used term in the title or the abstract | 2 |
|  |  | (*b*) Provide in the abstract an informative and balanced summary of what was done and what was found | 2 |
| Introduction | | |  |
| Background/rationale | 2 | Explain the scientific background and rationale for the investigation being reported | 4-5 |
| Objectives | 3 | State specific objectives, including any prespecified hypotheses | 5 |
| Methods | | |  |
| Study design | 4 | Present key elements of study design early in the paper | 6-7 |
| Setting | 5 | Describe the setting, locations, and relevant dates, including periods of recruitment, exposure, follow-up, and data collection | 6-7 |
| Participants | 6 | (*a*) Give the eligibility criteria, and the sources and methods of selection of participants | 6-7 |
| Variables | 7 | Clearly define all outcomes, exposures, predictors, potential confounders, and effect modifiers. Give diagnostic criteria, if applicable | 7-8 and Table 1 |
| Data sources/ measurement | 8* | For each variable of interest, give sources of data and details of methods of assessment (measurement). Describe comparability of assessment methods if there is more than one group | Table 1 |
| Bias | 9 | Describe any efforts to address potential sources of bias | 7 |
| Study size | 10 | Explain how the study size was arrived at | 7 and 17 |
| Quantitative variables | 11 | Explain how quantitative variables were handled in the analyses. If applicable, describe which groupings were chosen and why | 7-8 and Table 1 |
| Statistical methods | 12 | (*a*) Describe all statistical methods, including those used to control for confounding | 9 |
|  |  | (*b*) Describe any methods used to examine subgroups and interactions | 9 |
|  |  | (*c*) Explain how missing data were addressed | 9 |
|  |  | (*d*) If applicable, describe analytical methods taking account of sampling strategy |  |
|  |  | (*e*) Describe any sensitivity analyses | 9 |
| Results | | |  |
| Participants | 13* | (a) Report numbers of individuals at each stage of study—eg numbers potentially eligible, examined for eligibility, confirmed eligible, included in the study, completing follow-up, and analysed | 10 |
|  |  | (b) Give reasons for non-participation at each stage | 10 |
|  |  | (c) Consider use of a flow diagram |  |
| Descriptive data | 14* | (a) Give characteristics of study participants (eg demographic, clinical, social) and information on exposures and potential confounders | Table 2 |
|  |  | (b) Indicate number of participants with missing data for each variable of interest | Table 2 |
| Outcome data | 15* | Report numbers of outcome events or summary measures | Tables 3-4 |
| Main results | 16 | (*a*) Give unadjusted estimates and, if applicable, confounder-adjusted estimates and their precision (eg, 95% confidence interval). Make clear which confounders were adjusted for and why they were included | Tables 5-9 |
|  |  | (*b*) Report category boundaries when continuous variables were categorized | Table 2 |
|  |  | (*c*) If relevant, consider translating estimates of relative risk into absolute risk for a meaningful time period |  |
| Other analyses | 17 | Report other analyses done—eg analyses of subgroups and interactions, and sensitivity analyses | 12 |
| Discussion | | |  |
| Key results | 18 | Summarise key results with reference to study objectives | 15 |
| Limitations | 19 | Discuss limitations of the study, taking into account sources of potential bias or imprecision. Discuss both direction and magnitude of any potential bias | 17-18 |
| Interpretation | 20 | Give a cautious overall interpretation of results considering objectives, limitations, multiplicity of analyses, results from similar studies, and other relevant evidence | 15-16 |
| Generalisability | 21 | Discuss the generalisability (external validity) of the study results | 15-17 |
| Other information | | |  |
| Funding | 22 | Give the source of funding and the role of the funders for the present study and, if applicable, for the original study on which the present article is based | 20 |

*Give information separately for exposed and unexposed groups.

Table A2. Regression results for overall barrier and facilitator scores as well as unmet healthcare needs

| ***Independent variables*** | ***Overall barrier  score*** *Coefficient [95% CI]* | ***Overall facilitator  score*** *Coefficient [95% CI]* | ***Overall unmet healthcare needs*** *Odds ratio [95% CI]* |
| --- | --- | --- | --- |
| Female gender | -1.632  [-5.416, 2.152] | -0.064  [-0.909, 0.78] | 0.796  [0.400, 1.584] |
| *Aged 18-30 (base case)* |  |  |  |
| Aged 31-40 | 0.577  [-3.854, 5.008] | -0.280 [-1.269, 0.709] | 0.568  [0.251, 1.285] |
| Aged 41-50 | 0.230  [-4.069, 4.528] | -0.342  [-1.301, 0.618] | 0.852  [0.381, 1.905] |
| Aged 51-60 | -2.015  [-6.615, 2.584] | -0.716  [-1.743, 0.31] | 0.658  [0.279, 1.549] |
| Older than 60 | -7.605  [-15.87, 0.66] | 1.642  [-0.202, 3.487] | 0.548  [0.134, 2.244] |
| Living in a rural area | **3.005*  [0.061, 5.948]** | 0.394  [-0.263, 1.051] | **1.786*  [1.032, 3.091]** |
| Medical proximity | -1.68  [-4.678, 1.318] | -0.067  [-0.736, 0.602] | 1.366  [0.78, 2.39] |
| Complementary health insurance | **-3.233*  [-6.411, -0.054]** | 0.375  [-0.334, 1.084] | 0.933  [0.527, 1.651] |
| Household financial situation  good or very good | **-11.090***  [-14.854, -7.326]** | -0.412  [-1.252, 0.428] | 0.538  [0.287, 1.008] |
| *Household financial situation*  *medium (base case)* |  |  |  |
| Household financial situation  bad or very bad | **5.871***  [2.715, 9.028]** | -0.139  [-0.843, 0.566] | 1.473 [0.809, 2.684] |
| Living in a state with free central coordination services | **-5.318**  [-8.536, -2.1]** | -0.596  [-1.314, 0.122] | 0.639  [0.364, 1.123] |
| *Education below A-level  equivalent (base case)* |  |  |  |
| Education at A-level equivalent | 2.898  [-1.596, 7.392] | 0.597  [-0.406, 1.599] | 1.143  [0.508, 2.572] |
| Education higher than A-level  equivalent | 0.999  [-2.664, 4.662] | 0.512  [-0.306, 1.329] | 1.722  [0.871, 3.404] |
| Migration background | -3.322  [-7.716, 1.072] | 0.968  [-0.012, 1.949] | 1.865 [0.75, 4.636] |
| Living in a relationship | -0.889  [-3.887, 2.108] | 0.645  [-0.024, 1.314] | 0.943  [0.545, 1.632] |
| One or more physical chronic conditions | 0.432  [-2.489, 3.353] | 0.299  [-0.353, 0.951] | 1.390 [0.802, 2.407] |
| One or more mental health conditions | **-4.544**  [-7.926, -1.162]** | -0.461  [-1.215, 0.294] | 0.627  [0.345, 1.138] |
| Number of perceived severe symptoms | **1.849***  [1.222, 2.477]** | -0.031  [-0.171, 0.109] | **1.184**  [1.054, 1.331]** |
| Long COVID-19 onset | **1.521***  [0.622, 2.42]** | -0.016  [-0.216, 0.185] | **1.174*  [1.009, 1.365]** |
| Long COVID-19 duration | **0.609***  [0.315, 0.904]** | 0.002  [-0.064, 0.068] | **1.069**  [1.017, 1.123]** |
| Constant | 12.649  [-6.165, 31.463] | **7.400***  [3.201, 11.599]** | **0.031*  [0.001, 0.783]** |
| N | 387 | 387 | 387 |
| *Red numbers refer to adverse patient experiences, green to beneficial ones* * p-value < 0.05, ** p-value < 0.01, *** p-value < 0.001 | |  |  |

Table A3. Regression results for individual barriers by ‘access to care’ dimension (Levesque et al. 2013) for adult long COVID-19 patients in Austria

|  | ***Approachability*** | | | ***Acceptability (I/II)*** | | | |
| --- | --- | --- | --- | --- | --- | --- | --- |
|  | Too little available information about the condition and treatment options | Difficulties to assess the credibility of available information | Difficulties to perceive symptoms as unusual and to attribute them | GPs did not take me and my condition seriously | Specialists did not take me and my condition seriously | General practitioners attributed my symptoms to mental health problems | Specialists attributed my symptoms to mental health problems |
| ***Independent variables*** | *Odds ratio [95% CI]* | *Odds ratio [95% CI]* | *Odds ratio [95% CI]* | *Odds ratio [95% CI]* | *Odds ratio [95% CI]* | *Odds ratio [95% CI]* | *Odds ratio [95% CI]* |
| Female gender | 0.588 [0.288, 1.2] | 1.179 [0.687, 2.022] | 1.11 [0.642, 1.92] | 1.076 [0.609, 1.898] | 0.997 [0.575, 1.728] | 1.017 [0.576, 1.798] | 0.991 [0.567, 1.733] |
| *Aged 18-30 (base case)* |  |  |  |  |  |  |  |
| Aged 31-40 | 0.77 [0.366, 1.619] | 1.076 [0.571, 2.031] | 0.975 [0.51, 1.862] | 0.929 [0.495, 1.745] | 0.666 [0.341, 1.301] | 0.855 [0.453, 1.617] | 0.911 [0.478, 1.738] |
| Aged 41-50 | 1.063 [0.512, 2.208] | 1.335 [0.721, 2.472] | 0.748 [0.402, 1.392] | 0.905 [0.489, 1.673] | 0.53 [0.273, 1.032] | 1.023 [0.551, 1.9] | 0.661 [0.351, 1.246] |
| Aged 51-60 | 1.227 [0.55, 2.738] | 1.759 [0.899, 3.442] | 1.303 [0.664, 2.557] | 0.981 [0.502, 1.919] | 0.79 [0.384, 1.624] | 0.775 [0.393, 1.525] | 1.026 [0.518, 2.031] |
| Older than 60 | 0.803 [0.216, 2.985] | 2.989 [0.847, 10.553] | 1.315 [0.38, 4.546] | 0.825 [0.255, 2.669] | 0.46 [0.128, 1.653] | 0.611 [0.191, 1.953] | 1.144 [0.320, 4.090] |
| Living in a rural area | **1.907* [1.142, 3.183]** | **2.257*** [1.47, 3.466]** | 1.018 [0.669, 1.55] | 1.128 [0.734, 1.735] | 0.963 [0.618, 1.501] | 1.084 [0.699, 1.68] | 1.105 [0.717, 1.703] |
| Medical proximity | 0.805 [0.481, 1.346] | 0.795 [0.51, 1.24] | 0.784 [0.511, 1.202] | 0.825 [0.533, 1.275] | 1.367 [0.872, 2.143] | 0.822 [0.526, 1.285] | 1.151 [0.748, 1.77] |
| Complementary health insurance | **0.535* [0.317, 0.903]** | 0.986 [0.624, 1.559] | 0.955 [0.603, 1.513] | 0.711 [0.449, 1.126] | **0.608* [0.376, 0.981]** | 0.697 [0.431, 1.128] | 0.713 [0.446, 1.138] |
| Household financial situation good or very good | 0.665 [0.362, 1.224] | **0.499* [0.287, 0.869]** | 0.884 [0.518, 1.508] | **0.297*** [0.173, 0.509]** | 0.594 [0.344, 1.026] | **0.564* [0.323, 0.983]** | 0.911 [0.527, 1.574] |
| *Household financial situation* *medium (base case)* |  |  |  |  |  |  |  |
| Household financial situation bad or very bad | **1.992* [1.124, 3.533]** | 1.268 [0.802, 2.004] | 1.41 [0.895, 2.221] | 1.081 [0.679, 1.722] | 1.15 [0.712, 1.858] | 1.445 [0.906, 2.304] | 1.477 [0.929, 2.349] |
| Living in a state with central coordination | 1.029 [0.586, 1.808] | 1.49 [0.927, 2.395] | 0.89 [0.555, 1.427] | 1.000 [0.613, 1.633] | 0.741 [0.454, 1.209] | 0.983 [0.594, 1.626] | 0.988 [0.612, 1.595] |
| *Education below A-level equivalent (base case)* |  |  |  |  |  |  |  |
| Education at A-level equivalent | 1.513 [0.657, 3.485] | 1.183 [0.609, 2.297] | 1.388 [0.726, 2.652] | 0.888 [0.453, 1.744] | 1.985 [0.982, 4.012] | 0.77 [0.391, 1.518] | 1.765 [0.892, 3.491] |
| Education higher than A-level equivalent | 1.012 [0.534, 1.918] | **0.585* [0.343, 0.996]** | 0.763 [0.454, 1.28] | 0.939 [0.548, 1.609] | 1.039 [0.604, 1.788] | 0.773 [0.445, 1.341] | 0.973 [0.568, 1.668] |
| Migration background | 0.744 [0.358, 1.547] | 1.627 [0.839, 3.155] | 1.93 [0.988, 3.77] | 0.844 [0.44, 1.618] | 1.017 [0.512, 2.019] | 0.632 [0.321, 1.242] | 0.815 [0.416, 1.598] |
| Living in a relationship | 1.365 [0.814, 2.289] | 0.966 [0.623, 1.500] | 0.961 [0.621, 1.49] | 0.874 [0.561, 1.363] | 0.784 [0.494, 1.245] | 0.706 [0.454, 1.1] | 0.851 [0.547, 1.324] |
| One or more physical chronic conditions | 0.752 [0.457, 1.237] | **0.590* [0.387, 0.899]** | 0.752 [0.493, 1.146] | 0.791 [0.518, 1.209] | 0.823 [0.533, 1.271] | 0.98 [0.632, 1.522] | 0.871 [0.562, 1.351] |
| One or more mental health conditions | **0.518* [0.296, 0.904]** | 0.656 [0.401, 1.075] | 1.292 [0.792, 2.108] | 1.211 [0.73, 2.01] | 0.664 [0.403, 1.093] | 1.089 [0.668, 1.777] | 0.912 [0.553, 1.503] |
| Number of perceived severe symptoms | 1.024 [0.917, 1.143] | 1.034 [0.944, 1.133] | 1.012 [0.925, 1.107] | 1.047 [0.957, 1.145] | **1.106* [1.009, 1.213]** | **1.118* [1.019, 1.228]** | **1.114* [1.017, 1.221]** |
| Long COVID-19 onset | **1.191* [1.04, 1.364]** | 1.002 [0.877, 1.145] | 1.025 [0.904, 1.162] | 1.092 [0.971, 1.228] | 1.042 [0.903, 1.203] | 1.111 [0.955, 1.294] | 1.047 [0.894, 1.227] |
| Long COVID-19 duration | **1.066** [1.02, 1.114]** | 0.996 [0.953, 1.04] | 0.999 [0.959, 1.04] | **1.050* [1.011, 1.092]** | 1.046 [0.998, 1.097] | 1.055* [1.002, 1.11] | 1.05 [0.996, 1.106] |
| N | 384 | 383 | 381 | 379 | 380 | 354 | 366 |
| *Red numbers refer to adverse patient experiences, green to beneficial ones; N varies by barrier as only applicable participants are included in the analysis* * p-value < 0.05, ** p-value < 0.01, *** p-value < 0.001 | | | | | | | |

|  | ***Acceptability (II/II)*** | | | | | ***Availability & accommodation (I/III)*** | |
| --- | --- | --- | --- | --- | --- | --- | --- |
|  | Doctors or assessors ignored the findings of other medical staff or did not take them seriously | Friends or family did not take my condition seriously | My professional environment did not take my condition seriously | I questioned myself as to whether I was really ill | I did not know my way around the health system and did not know what to look for | Outpatient clinics or resident specialists required a referral from other doctors or certain diagnoses | I had to see doctors more often than necessary to provide my health insurance with ongoing evidence of my condition |
| ***Independent variables*** | *Odds ratio [95% CI]* | *Odds ratio [95% CI]* | *Odds ratio [95% CI]* | *Odds ratio [95% CI]* | *Odds ratio [95% CI]* | *Odds ratio [95% CI]* | *Odds ratio [95% CI]* |
| Female gender | 1.001 [0.508, 1.972] | 0.667 [0.387, 1.15] | 0.968 [0.552, 1.7] | 0.99 [0.594, 1.649] | 1.465 [0.825, 2.602] | 0.987 [0.578, 1.688] | 0.926 [0.513, 1.67] |
| *Aged 18-30 (base case)* |  |  |  |  |  |  |  |
| Aged 31-40 | 1.585 [0.735, 3.416] | 0.776 [0.418, 1.438] | 1.092 [0.57, 2.093] | 1.544 [0.817, 2.917] | 0.838 [0.427, 1.647] | 1.083 [0.559, 2.098] | 1.883 [0.924, 3.838] |
| Aged 41-50 | 1.895 [0.918, 3.911] | 1.164 [0.647, 2.091] | 1.812 [0.955, 3.437] | 0.898 [0.488, 1.654] | 0.953 [0.498, 1.824] | 1.304 [0.682, 2.496] | 1.703 [0.851, 3.407] |
| Aged 51-60 | 1.491 [0.671, 3.314] | 1.673 [0.883, 3.17] | **2.515** [1.271, 4.978]** | 1.017 [0.515, 2.006] | 0.838 [0.413, 1.701] | 1.342 [0.666, 2.703] | 1.581 [0.742, 3.369] |
| Older than 60 | 2.856 [0.517, 15.78] | 0.809 [0.245, 2.665] | 1.186 [0.236, 5.951] | 0.529 [0.18, 1.554] | 0.745 [0.219, 2.536] | 0.929 [0.275, 3.133] | 1.37 [0.3, 6.26] |
| Living in a rural area | 0.905 [0.53, 1.546] | 1.347 [0.89, 2.039] | 1.255 [0.812, 1.939] | 1.199 [0.787, 1.826] | **1.603* [1.009, 2.549]** | 0.877 [0.569, 1.353] | 1.32 [0.82, 2.125] |
| Medical proximity | 1.13 [0.652, 1.957] | 0.805 [0.526, 1.233] | 1.452 [0.927, 2.274] | 0.85 [0.554, 1.306] | **0.200*** [0.12, 0.332]** | 0.903 [0.581, 1.403] | 0.862 [0.529, 1.407] |
| Complementary health insurance | 0.971 [0.557, 1.692] | 0.824 [0.528, 1.287] | 0.988 [0.617, 1.582] | 0.829 [0.523, 1.315] | **0.419** [0.249, 0.704]** | 0.695 [0.436, 1.109] | **0.523* [0.315, 0.867]** |
| Household financial situation good or very good | **0.336*** [0.177, 0.639]** | 0.65 [0.379, 1.114] | **0.417** [0.239, 0.728]** | 1.147 [0.677, 1.942] | 0.599 [0.335, 1.071] | 0.73 [0.418, 1.278] | **0.515* [0.279, 0.953]** |
| *Household financial situation* *medium (base case)* |  |  |  |  |  |  |  |
| Household financial situation bad or very bad | **2.546** [1.438, 4.51]** | 1.173 [0.751, 1.833] | 0.91 [0.569, 1.456] | 1.313 [0.828, 2.082] | 1.158 [0.715, 1.878] | 1.47 [0.926, 2.332] | 1.403 [0.844, 2.333] |
| Living in a state with central coordination | **0.451** [0.256, 0.796]** | 0.766 [0.487, 1.205] | 1.068 [0.658, 1.736] | 0.919 [0.576, 1.465] | 1.325 [0.811, 2.165] | 0.751 [0.472, 1.193] | 0.813 [0.488, 1.353] |
| *Education below A-level equivalent (base case)* |  |  |  |  |  |  |  |
| Education at A-level equivalent | 0.931 [0.401, 2.162] | 1.144 [0.611, 2.143] | 0.733 [0.365, 1.473] | 1.451 [0.751, 2.803] | 0.907 [0.457, 1.802] | 1.709 [0.893, 3.271] | 0.776 [0.377, 1.597] |
| Education higher than A-level equivalent | 0.637 [0.328, 1.238] | 1.138 [0.68, 1.905] | 0.805 [0.46, 1.408] | 0.808 [0.476, 1.371] | 0.815 [0.47, 1.413] | 1.51 [0.887, 2.57] | 1.242 [0.684, 2.257] |
| Migration background | 0.594 [0.284, 1.242] | 1.204 [0.643, 2.256] | 0.785 [0.413, 1.493] | 1.176 [0.641, 2.158] | 1.377 [0.671, 2.827] | 0.633 [0.333, 1.204] | **0.434* [0.216, 0.872]** |
| Living in a relationship | 0.979 [0.568, 1.685] | 0.966 [0.632, 1.476] | 0.809 [0.514, 1.272] | 1.477 [0.951, 2.293] | 0.772 [0.484, 1.231] | 0.841 [0.537, 1.316] | 0.778 [0.472, 1.284] |
| One or more physical chronic conditions | 1.098 [0.654, 1.845] | 1.357 [0.898, 2.052] | 0.943 [0.61, 1.46] | 0.808 [0.531, 1.228] | **0.609* [0.386, 0.962]** | 0.893 [0.587, 1.36] | 1.26 [0.781, 2.033] |
| One or more mental health conditions | 1.22 [0.623, 2.391] | 0.946 [0.588, 1.525] | 0.68 [0.404, 1.145] | **1.837* [1.137, 2.968]** | 1.156 [0.689, 1.942] | 0.718 [0.437, 1.18] | 0.941 [0.542, 1.633] |
| Number of perceived severe symptoms | 1.101 [0.983, 1.233] | 1.048 [0.959, 1.145] | **1.129* [1.025, 1.244]** | 1.028 [0.939, 1.125] | 0.964 [0.876, 1.061] | **1.182*** [1.08, 1.295]** | **1.171** [1.057, 1.297]** |
| Long COVID-19 onset | 1.051 [0.9, 1.227] | 1.007 [0.896, 1.131] | 1.029 [0.916, 1.156] | 0.871 [0.753, 1.006] | 1.07 [0.923, 1.24] | 0.978 [0.849, 1.127] | **1.291** [1.103, 1.512]** |
| Long COVID-19 duration | **1.054* [1.001, 1.11]** | 1.021 [0.983, 1.061] | 1.028 [0.99, 1.068] | **0.944* [0.9, 0.989]** | 1.02 [0.972, 1.07] | 1.002 [0.956, 1.05] | **1.092*** [1.036, 1.15]** |
| N | 325 | 383 | 353 | 368 | 339 | 354 | 334 |
| *Red numbers refer to adverse patient experiences, green to beneficial ones; N varies by barrier as only applicable participants are included in the analysis* * p-value < 0.05, ** p-value < 0.01, *** p-value < 0.001 | | | | | | | |

|  | ***Availability & accommodation (II/III)*** | | | | | | |
| --- | --- | --- | --- | --- | --- | --- | --- |
|  | GPs did not accept new patients or only had open appointments in the distant future | Resident specialists did not accept new patients or only had open appointments in the distant future | Hospital outpatient clinics incl. long COVID-19 clinics did not  accept new patients or only had distant-future appointments | GPs didn't take enough time for me | Specialists didn't take enough time for me | Too little time was taken for me in the hospital outpatient clinic | Long COVID-19 outpatient clinics were closed before my treatment was completed/started |
| ***Independent variables*** | *Odds ratio [95% CI]* | *Odds ratio [95% CI]* | *Odds ratio [95% CI]* | *Odds ratio [95% CI]* | *Odds ratio [95% CI]* | *Odds ratio [95% CI]* | *Odds ratio [95% CI]* |
| Female gender | 0.704 [0.373, 1.326] | 1.108 [0.615, 1.997] | 1.103 [0.566, 2.149] | 1.605 [0.912, 2.823] | 1.146 [0.663, 1.982] | 0.649 [0.324, 1.301] | 1.024 [0.451, 2.327] |
| *Aged 18-30 (base case)* |  |  |  |  |  |  |  |
| Aged 31-40 | 1.375 [0.614, 3.079] | 0.909 [0.432, 1.914] | 1.475 [0.656, 3.313] | 0.809 [0.437, 1.497] | 1.003 [0.533, 1.886] | 0.746 [0.321, 1.736] | 1.069 [0.407, 2.808] |
| Aged 41-50 | 1.86 [0.863, 4.011] | 0.643 [0.312, 1.323] | 1.151 [0.541, 2.447] | 0.86 [0.475, 1.558] | 0.707 [0.38, 1.314] | 0.863 [0.375, 1.984] | 1.698 [0.634, 4.545] |
| Aged 51-60 | 1.283 [0.548, 2.999] | 0.534 [0.25, 1.141] | 0.984 [0.423, 2.289] | 1.035 [0.541, 1.982] | 0.968 [0.49, 1.914] | 0.74 [0.296, 1.85] | 1.1 [0.379, 3.187] |
| Older than 60 | 0.985 [0.172, 5.634] | 0.546 [0.152, 1.96] | 1.936 [0.306, 12.242] | 0.532 [0.16, 1.773] | 0.706 [0.205, 2.432] | 3.512 [0.489, 25.249] | 0.981 [0.138, 6.986] |
| Living in a rural area | 0.697 [0.413, 1.176] | 0.771 [0.486, 1.223] | 1.306 [0.753, 2.268] | 1.264 [0.831, 1.921] | 0.877 [0.574, 1.339] | 0.965 [0.556, 1.675] | 0.963 [0.484, 1.917] |
| Medical proximity | **0.518* [0.295, 0.91]** | 0.807 [0.505, 1.29] | 1.072 [0.602, 1.907] | 0.815 [0.531, 1.252] | 1.337 [0.869, 2.056] | 0.871 [0.489, 1.55] | 0.849 [0.416, 1.734] |
| Complementary health insurance | 0.709 [0.398, 1.265] | **0.430*** [0.263, 0.704]** | 0.705 [0.394, 1.259] | 0.857 [0.541, 1.357] | 0.722 [0.458, 1.138] | 0.807 [0.453, 1.437] | 1.024 [0.472, 2.223] |
| Household financial situation good or very good | 0.752 [0.359, 1.572] | 0.588 [0.328, 1.055] | 0.663 [0.329, 1.333] | **0.434** [0.252, 0.745]** | **0.544* [0.315, 0.94]** | **0.387* [0.181, 0.829]** | **0.308* [0.126, 0.755]** |
| *Household financial situation* *medium (base case)* |  |  |  |  |  |  |  |
| Household financial situation bad or very bad | 1.574 [0.901, 2.748] | 0.912 [0.548, 1.519] | **1.886* [1.048, 3.395]** | 1.287 [0.82, 2.022] | 1.44 [0.916, 2.266] | 1.073 [0.592, 1.945] | 1.166 [0.532, 2.556] |
| Living in a state with central coordination | 1.676 [0.953, 2.949] | **0.335*** [0.204, 0.547]** | **0.242*** [0.137, 0.426]** | 0.795 [0.499, 1.267] | 0.803 [0.5, 1.29] | **0.444** [0.248, 0.794]** | **0.204*** [0.097, 0.425]** |
| *Education below A-level equivalent (base case)* |  |  |  |  |  |  |  |
| Education at A-level equivalent | 1.36 [0.624, 2.962] | 1.601 [0.778, 3.295] | 1.131 [0.516, 2.48] | 1.025 [0.533, 1.974] | **2.464** [1.264, 4.802]** | 1.155 [0.507, 2.63] | 0.646 [0.215, 1.94] |
| Education higher than A-level equivalent | 1.031 [0.538, 1.974] | 1.025 [0.575, 1.826] | 1.538 [0.775, 3.049] | 1.12 [0.663, 1.894] | 1.19 [0.697, 2.031] | 1.099 [0.548, 2.204] | 1.029 [0.416, 2.544] |
| Migration background | 1.089 [0.502, 2.366] | 0.606 [0.308, 1.19] | 0.72 [0.315, 1.645] | 1.29 [0.675, 2.465] | **0.505* [0.263, 0.968]** | 1.353 [0.541, 3.381] | 1.185 [0.413, 3.405] |
| Living in a relationship | 0.833 [0.48, 1.444] | 0.874 [0.534, 1.431] | 1.262 [0.708, 2.25] | 0.9 [0.585, 1.386] | 0.717 [0.463, 1.112] | 0.681 [0.383, 1.212] | 0.571 [0.272, 1.199] |
| One or more physical chronic conditions | 1.008 [0.592, 1.715] | 1.075 [0.674, 1.715] | 1.156 [0.659, 2.025] | 1 [0.657, 1.521] | 1.087 [0.715, 1.654] | 1.303 [0.74, 2.295] | 1.329 [0.65, 2.716] |
| One or more mental health conditions | **0.387** [0.194, 0.772]** | 0.766 [0.451, 1.303] | 0.75 [0.396, 1.419] | 0.842 [0.512, 1.383] | 0.79 [0.481, 1.298] | 0.762 [0.397, 1.461] | 0.598 [0.257, 1.391] |
| Number of perceived severe symptoms | **1.174** [1.045, 1.319]** | **1.129* [1.022, 1.247]** | 1.019 [0.908, 1.143] | 1.065 [0.975, 1.163] | 1.088 [0.993, 1.193] | **1.142* [1.014, 1.286]** | 1.161 [0.997, 1.352] |
| Long COVID-19 onset | 1.299 [0.959, 1.759] | **1.233** [1.063, 1.43]** | **1.274** [1.076, 1.507]** | **1.181* [1.012, 1.378]** | 1.044 [0.909, 1.199] | 1.067 [0.874, 1.304] | 1.26 [0.925, 1.718] |
| Long COVID-19 duration | 1.082 [0.979, 1.195] | **1.064* [1.014, 1.118]** | 1.055 [0.999, 1.115] | 1.05 [0.998, 1.105] | 1.026 [0.98, 1.073] | 1.016 [0.951, 1.085] | 1.068 [0.966, 1.18] |
| N | 319 | 370 | 303 | 369 | 371 | 217 | 200 |
| *Red numbers refer to adverse patient experiences, green to beneficial ones; N varies by barrier as only applicable participants are included in the analysis* * p-value < 0.05, ** p-value < 0.01, *** p-value < 0.001 | | | | | | | |

|  | ***Availability & accommodation (III/III)*** | | | | | | |
| --- | --- | --- | --- | --- | --- | --- | --- |
|  | The facilities in the waiting areas of the doctors I visited did not meet my needs (e.g., too loud, too bright) | Travelling to my GP was a burden (e.g., far away, poor public transport options) | Travelling to relevant specialists was a burden (e.g., far away, poor public transport options) | Travelling to relevant hospital outpatient clinics was a burden (e.g., far away, public transport options) | Telemedicine or home visits were not offered by GPs, although I would have needed them | Telemedicine or home visits were not offered by specialists, although I would have needed them | Organising my treat-ments or administrative procedures myself was a burden (e.g., making appointments) |
| ***Independent variables*** | *Odds ratio [95% CI]* | *Odds ratio [95% CI]* | *Odds ratio [95% CI]* | *Odds ratio [95% CI]* | *Odds ratio [95% CI]* | *Odds ratio [95% CI]* | *Odds ratio [95% CI]* |
| Female gender | **2.078* [1.176, 3.672]** | 1.127 [0.648, 1.958] | 1.554 [0.876, 2.758] | 0.96 [0.468, 1.966] | 0.658 [0.327, 1.323] | 0.699 [0.348, 1.405] | 0.833 [0.418, 1.661] |
| *Aged 18-30 (base case)* |  |  |  |  |  |  |  |
| Aged 31-40 | 0.728 [0.365, 1.449] | 0.905 [0.475, 1.724] | 1.039 [0.509, 2.121] | 0.823 [0.346, 1.959] | 1.747 [0.793, 3.848] | 1.157 [0.545, 2.457] | 1.308 [0.613, 2.791] |
| Aged 41-50 | 0.849 [0.427, 1.686] | 0.889 [0.469, 1.684] | 0.662 [0.331, 1.324] | 1.181 [0.497, 2.81] | 1.422 [0.674, 3] | 1.033 [0.507, 2.104] | 1.928 [0.903, 4.116] |
| Aged 51-60 | 0.571 [0.279, 1.168] | **0.449* [0.225, 0.898]** | 0.556 [0.267, 1.159] | 0.785 [0.3, 2.054] | 0.878 [0.385, 2.003] | 0.582 [0.263, 1.29] | 1.171 [0.537, 2.552] |
| Older than 60 | **0.238* [0.065, 0.878]** | 1.273 [0.372, 4.362] | 0.385 [0.12, 1.234] | 0.254 [0.039, 1.669] | 1.549 [0.295, 8.122] | 0.52 [0.082, 3.297] | 0.52 [0.132, 2.043] |
| Living in a rural area | 1.211 [0.775, 1.894] | 1.362 [0.884, 2.099] | **2.256*** [1.408, 3.613]** | **2.881*** [1.588, 5.225]** | 1.537 [0.89, 2.652] | 1.606 [0.954, 2.703] | 1.547 [0.907, 2.64] |
| Medical proximity | 0.792 [0.504, 1.246] | 0.985 [0.632, 1.536] | 0.856 [0.533, 1.374] | 0.923 [0.512, 1.666] | 0.933 [0.531, 1.637] | 0.766 [0.449, 1.305] | 0.816 [0.475, 1.399] |
| Complementary health insurance | 1.153 [0.708, 1.878] | 0.954 [0.594, 1.533] | 0.736 [0.451, 1.202] | 0.687 [0.374, 1.262] | 0.843 [0.472, 1.505] | 0.583 [0.337, 1.008] | 0.68 [0.39, 1.185] |
| Household financial situation good or very good | **0.490* [0.278, 0.862]** | **0.437** [0.247, 0.773]** | 0.685 [0.388, 1.211] | 0.7 [0.329, 1.488] | **0.455* [0.218, 0.953]** | 0.577 [0.281, 1.185] | **0.494* [0.264, 0.925]** |
| *Household financial situation* *medium (base case)* |  |  |  |  |  |  |  |
| Household financial situation bad or very bad | 1.486 [0.914, 2.414] | 1.178 [0.736, 1.885] | 1.213 [0.727, 2.025] | 1.301 [0.685, 2.472] | 1.349 [0.768, 2.369] | 1.223 [0.707, 2.115] | 1.279 [0.71, 2.304] |
| Living in a state with central coordination | 1.07 [0.656, 1.744] | 0.721 [0.447, 1.164] | **0.367*** [0.222, 0.606]** | **0.318*** [0.171, 0.591]** | 0.718 [0.4, 1.291] | 1.095 [0.618, 1.942] | 0.701 [0.4, 1.229] |
| *Education below A-level equivalent (base case)* |  |  |  |  |  |  |  |
| Education at A-level equivalent | 1.859 [0.923, 3.744] | 1.089 [0.548, 2.163] | 0.926 [0.436, 1.964] | 0.879 [0.363, 2.128] | 0.432 [0.184, 1.015] | 0.911 [0.402, 2.062] | 1.639 [0.697, 3.852] |
| Education higher than A-level equivalent | 1.371 [0.787, 2.39] | 1.494 [0.864, 2.583] | 0.775 [0.430, 1.400] | 0.936 [0.435, 2.015] | 0.707 [0.351, 1.422] | 0.916 [0.477, 1.757] | 1.256 [0.643, 2.454] |
| Migration background | 1.068 [0.549, 2.08] | 1.115 [0.567, 2.191] | 1.025 [0.504, 2.084] | 0.933 [0.39, 2.235] | 0.761 [0.341, 1.701] | **0.360* [0.162, 0.804]** | 1.083 [0.491, 2.387] |
| Living in a relationship | 1.229 [0.775, 1.949] | 0.882 [0.56, 1.388] | 1.114 [0.693, 1.792] | 0.749 [0.409, 1.375] | **0.464** [0.263, 0.818]** | 0.876 [0.517, 1.482] | 1.069 [0.625, 1.827] |
| One or more physical chronic conditions | 0.856 [0.551, 1.331] | 1.283 [0.835, 1.971] | 0.881 [0.555, 1.4] | 0.804 [0.445, 1.454] | 1.046 [0.606, 1.807] | 1.164 [0.684, 1.981] | 1.072 [0.63, 1.825] |
| One or more mental health conditions | 0.785 [0.465, 1.325] | 0.643 [0.394, 1.049] | 0.848 [0.499, 1.442] | **0.424* [0.215, 0.84]** | 0.835 [0.439, 1.588] | 0.814 [0.45, 1.473] | 0.917 [0.499, 1.687] |
| Number of perceived severe symptoms | **1.260*** [1.136, 1.397]** | **1.216*** [1.102, 1.343]** | **1.214*** [1.096, 1.345]** | **1.259*** [1.104, 1.437]** | **1.173* [1.036, 1.328]** | **1.178** [1.048, 1.325]** | **1.201** [1.072, 1.347]** |
| Long COVID-19 onset | 1.101 [0.97, 1.25] | 1.107 [0.962, 1.273] | 1.072 [0.939, 1.223] | 1.122 [0.954, 1.319] | 1.105 [0.894, 1.366] | 1.034 [0.845, 1.267] | **1.224** [1.062, 1.41]** |
| Long COVID-19 duration | 1.018 [0.977, 1.061] | 1.017 [0.971, 1.065] | 1.027 [0.984, 1.073] | 1.034 [0.982, 1.09] | 1.028 [0.959, 1.102] | 0.997 [0.932, 1.065] | **1.064** [1.015, 1.114]** |
| N | 362 | 358 | 374 | 248 | 254 | 255 | 378 |
| *Red numbers refer to adverse patient experiences, green to beneficial ones; N varies by barrier as only applicable participants are included in the analysis* * p-value < 0.05, ** p-value < 0.01, *** p-value < 0.001 | | | | | | | |

|  | ***Affordability (I/II)*** | | | | | | |
| --- | --- | --- | --- | --- | --- | --- | --- |
|  | Costs for GPs posed a financial burden for me | Costs for specialists posed a financial burden for me | Costs for medication posed a financial burden for me | Costs for dietary supplements posed a financial burden for me | Costs for other treatments (e.g., physiotherapy, homeopathy) posed a financial burden for me | I had to weigh up different treatments against each other due to costs | I had to go to private doctors as I could not find insurance-covered doctors with long COVID-19 expertise |
| ***Independent variables*** | *Odds ratio [95% CI]* | *Odds ratio [95% CI]* | *Odds ratio [95% CI]* | *Odds ratio [95% CI]* | *Odds ratio [95% CI]* | *Odds ratio [95% CI]* | *Odds ratio [95% CI]* |
| Female gender | 0.557 [0.3, 1.033] | 0.706 [0.386, 1.29] | 0.743 [0.409, 1.351] | 1.315 [0.684, 2.528] | 1.418 [0.73, 2.755] | 1.289 [0.682, 2.436] | 1.382 [0.601, 3.179] |
| *Aged 18-30 (base case)* |  |  |  |  |  |  |  |
| Aged 31-40 | 1.074 [0.503, 2.293] | 0.731 [0.356, 1.504] | 1.111 [0.55, 2.241] | 1.567 [0.709, 3.465] | 1.24 [0.574, 2.678] | 1.876 [0.891, 3.95] | 0.538 [0.187, 1.546] |
| Aged 41-50 | 1.556 [0.749, 3.233] | 0.736 [0.365, 1.483] | 0.811 [0.411, 1.599] | 1.497 [0.704, 3.182] | 1.619 [0.766, 3.425] | 1.612 [0.796, 3.266] | 1.205 [0.4, 3.63] |
| Aged 51-60 | 0.824 [0.368, 1.845] | 0.485 [0.23, 1.024] | 0.784 [0.379, 1.621] | 0.93 [0.416, 2.081] | 0.928 [0.412, 2.09] | 1.559 [0.702, 3.462] | 0.406 [0.138, 1.198] |
| Older than 60 | 0.151 [0.016, 1.418] | 0.502 [0.137, 1.847] | 0.472 [0.14, 1.591] | 1.171 [0.276, 4.968] | 0.367 [0.104, 1.289] | **0.161* [0.04, 0.651]** | 0.432 [0.082, 2.267] |
| Living in a rural area | 1.495 [0.901, 2.481] | **1.655* [1.04, 2.633]** | 1.414 [0.897, 2.23] | 1.573 [0.927, 2.668] | 1.245 [0.733, 2.116] | 1.187 [0.719, 1.958] | 0.988 [0.505, 1.935] |
| Medical proximity | 0.796 [0.475, 1.334] | 0.719 [0.448, 1.153] | 0.936 [0.59, 1.487] | 0.978 [0.569, 1.679] | **0.581* [0.338, 0.999]** | 0.697 [0.412, 1.181] | 0.591 [0.295, 1.184] |
| Complementary health insurance | 0.788 [0.448, 1.384] | **0.502** [0.307, 0.821]** | 1.065 [0.649, 1.748] | 1.31 [0.743, 2.308] | 0.739 [0.428, 1.275] | 0.632 [0.376, 1.06] | **0.307*** [0.155, 0.607]** |
| Household financial situation good or very good | 0.488 [0.229, 1.042] | **0.293*** [0.164, 0.524]** | **0.365*** [0.204, 0.654]** | **0.266*** [0.144, 0.491]** | **0.313*** [0.171, 0.573]** | **0.252*** [0.136, 0.468]** | **0.290** [0.135, 0.62]** |
| *Household financial situation* *medium (base case)* |  |  |  |  |  |  |  |
| Household financial situation bad or very bad | **1.849* [1.091, 3.132]** | **2.812*** [1.683, 4.698]** | **3.313*** [2.003, 5.479]** | **5.503*** [2.948, 10.271]** | **3.787*** [2.042, 7.021]** | **3.348*** [1.893, 5.921]** | **2.803* [1.215, 6.463]** |
| Living in a state with central coordination | 1.193 [0.687, 2.073] | **0.494** [0.297, 0.822]** | **0.399*** [0.242, 0.658]** | **0.368*** [0.213, 0.636]** | **0.568* [0.324, 0.994]** | 0.95 [0.536, 1.685] | **0.221*** [0.112, 0.435]** |
| *Education below A-level equivalent (base case)* |  |  |  |  |  |  |  |
| Education at A-level equivalent | 0.476 [0.209, 1.083] | 0.859 [0.418, 1.767] | 1.362 [0.658, 2.819] | 1.281 [0.556, 2.953] | 1.053 [0.438, 2.533] | 0.677 [0.303, 1.512] | 1.529 [0.535, 4.374] |
| Education higher than A-level equivalent | 0.762 [0.412, 1.408] | 1.024 [0.574, 1.827] | 0.741 [0.419, 1.31] | 0.873 [0.446, 1.708] | 0.621 [0.309, 1.246] | 0.804 [0.418, 1.549] | 1.726 [0.75, 3.97] |
| Migration background | 1.118 [0.528, 2.368] | 1.187 [0.581, 2.423] | 1.032 [0.515, 2.069] | 1.003 [0.456, 2.208] | **0.364** [0.17, 0.782]** | 1.102 [0.508, 2.39] | 0.881 [0.299, 2.59] |
| Living in a relationship | **0.373*** [0.223, 0.622]** | 0.801 [0.49, 1.31] | 0.957 [0.592, 1.545] | 0.689 [0.392, 1.212] | 1.433 [0.824, 2.494] | 0.617 [0.355, 1.072] | 0.897 [0.427, 1.884] |
| One or more physical chronic conditions | 0.735 [0.439, 1.23] | 1.345 [0.844, 2.142] | 1.369 [0.872, 2.15] | 1.092 [0.653, 1.829] | 1.334 [0.79, 2.253] | 0.936 [0.57, 1.538] | 1.245 [0.62, 2.497] |
| One or more mental health conditions | **0.519* [0.277, 0.972]** | 0.642 [0.375, 1.099] | **0.437** [0.259, 0.739]** | **0.404** [0.224, 0.732]** | 0.612 [0.338, 1.107] | 0.595 [0.332, 1.068] | 0.508 [0.225, 1.146] |
| Number of perceived severe symptoms | 1.093 [0.977, 1.222] | 1.092 [0.986, 1.21] | **1.183** [1.07, 1.308]** | **1.151* [1.026, 1.291]** | **1.176** [1.05, 1.318]** | **1.217*** [1.089, 1.36]** | 1.087 [0.94, 1.258] |
| Long COVID-19 onset | 0.992 [0.848, 1.16] | 1.127 [0.991, 1.281] | 1.081 [0.934, 1.252] | **1.233** [1.057, 1.438]** | **1.248** [1.09, 1.428]** | **1.318*** [1.125, 1.545]** | **1.257** [1.06, 1.492]** |
| Long COVID-19 duration | 0.993 [0.943, 1.046] | 1.045* [1.003, 1.09] | 1.025 [0.977, 1.076] | 1.064* [1.011, 1.12] | 1.061** [1.017, 1.108] | 1.097*** [1.042, 1.156] | 1.078** [1.018, 1.141] |
| N | 343 | 369 | 366 | 365 | 360 | 352 | 349 |
| *Red numbers refer to adverse patient experiences, green to beneficial ones; N varies by barrier as only applicable participants are included in the analysis* * p-value < 0.05, ** p-value < 0.01, *** p-value < 0.001 | | | | | | | |

|  | ***Affordability (II/II)*** | ***Appropriateness (I/II)*** | | | | | |
| --- | --- | --- | --- | --- | --- | --- | --- |
|  | I had to go to elective or private doctors due to waiting times at doctors covered by statutory health insurance | My GP had difficulties diagnosing long COVID-19 | The specialists I consulted had difficulties diagnosing long COVID-19 | My GP had difficulties treating my symptoms | The specialists I consulted had difficulties treating my symptoms | I had difficulties receiving or extending sick pay | I had difficulties obtaining a state disability pension |
| ***Independent variables*** | *Odds ratio [95% CI]* | *Odds ratio [95% CI]* | *Odds ratio [95% CI]* | *Odds ratio [95% CI]* | *Odds ratio [95% CI]* | *Odds ratio [95% CI]* | *Odds ratio [95% CI]* |
| Female gender | 1.169 [0.614, 2.229] | 0.958 [0.53, 1.731] | 0.903 [0.528, 1.543] | 1.376 [0.683, 2.774] | 0.97 [0.55, 1.712] | 1.133 [0.614, 2.09] | 0.767 [0.189, 3.106] |
| *Aged 18-30 (base case)* |  |  |  |  |  |  |  |
| Aged 31-40 | 1.417 [0.662, 3.034] | **1.982* [1.031, 3.81]** | 1.261 [0.664, 2.397] | 1.196 [0.486, 2.944] | 0.523 [0.257, 1.068] | 0.901 [0.416, 1.953] | 1.16 [0.172, 7.805] |
| Aged 41-50 | 1.522 [0.726, 3.189] | 1.752 [0.936, 3.281] | **1.876* [1.002, 3.514]** | 1.002 [0.437, 2.297] | 0.648 [0.325, 1.29] | 0.843 [0.398, 1.788] | 1.592 [0.221, 11.468] |
| Aged 51-60 | 1.181 [0.524, 2.661] | **2.055* [1.028, 4.107]** | 1.514 [0.776, 2.953] | 0.558 [0.234, 1.328] | **0.424* [0.203, 0.884]** | 0.907 [0.408, 2.015] | 1.101 [0.156, 7.785] |
| Older than 60 | 0.975 [0.241, 3.94] | 3 [0.896, 10.042] | 1.966 [0.584, 6.617] | 0.536 [0.132, 2.178] | 0.709 [0.179, 2.802] | *n/a* | *n/a* |
| Living in a rural area | 1.402 [0.825, 2.38] | 0.962 [0.615, 1.504] | 1.012 [0.671, 1.528] | 1.131 [0.641, 1.995] | 1.05 [0.67, 1.646] | 0.994 [0.61, 1.62] | 0.81 [0.224, 2.932] |
| Medical proximity | 0.82 [0.478, 1.407] | 0.799 [0.506, 1.261] | 1.023 [0.674, 1.551] | 0.753 [0.421, 1.35] | 0.99 [0.632, 1.55] | 0.915 [0.559, 1.499] | 1.417 [0.441, 4.551] |
| Complementary health insurance | 0.581 [0.335, 1.01] | **0.614* [0.38, 0.99]** | **0.608* [0.385, 0.96]** | **0.391** [0.217, 0.707]** | 0.669 [0.412, 1.086] | 0.75 [0.433, 1.3] | 0.794 [0.241, 2.621] |
| Household financial situation good or very good | **0.441* [0.231, 0.843]** | **0.394** [0.226, 0.688]** | 0.977 [0.573, 1.665] | **0.322** [0.161, 0.646]** | 0.643 [0.365, 1.135] | **0.426* [0.211, 0.86]** | 0.412 [0.115, 1.471] |
| *Household financial situation* *medium (base case)* |  |  |  |  |  |  |  |
| Household financial situation bad or very bad | 1.217 [0.678, 2.183] | 0.894 [0.546, 1.462] | 0.982 [0.627, 1.538] | 0.826 [0.429, 1.594] | 0.904 [0.556, 1.47] | **2.840*** [1.71, 4.719]** | **7.593** [1.733, 33.275]** |
| Living in a state with central coordination | **0.352*** [0.194, 0.639]** | 0.9 [0.553, 1.464] | 0.778 [0.495, 1.224] | 0.727 [0.4, 1.32] | 1.013 [0.621, 1.654] | 0.595 [0.351, 1.007] | **0.182** [0.054, 0.611]** |
| *Education below A-level equivalent (base case)* |  |  |  |  |  |  |  |
| Education at A-level equivalent | 1.693 [0.728, 3.933] | 1.784 [0.882, 3.607] | **2.726** [1.394, 5.33]** | 1.448 [0.614, 3.416] | **3.000** [1.452, 6.2]** | 1.359 [0.655, 2.823] | 1.753 [0.173, 17.732] |
| Education higher than A-level equivalent | 1.04 [0.542, 1.994] | 1.739 [0.997, 3.033] | 1.411 [0.841, 2.365] | 1.887 [0.945, 3.771] | 1.699 [0.979, 2.949] | 0.992 [0.549, 1.794] | 0.201 [0.039, 1.027] |
| Migration background | 1.185 [0.523, 2.681] | 0.654 [0.335, 1.277] | 0.546 [0.286, 1.044] | 0.437* [0.195, 0.979] | **0.425* [0.219, 0.824]** | 0.548 [0.272, 1.102] | 0.558 [0.108, 2.895] |
| Living in a relationship | 1.242 [0.726, 2.124] | 0.885 [0.554, 1.413] | 0.938 [0.606, 1.451] | 1.195 [0.667, 2.142] | 0.772 [0.483, 1.235] | 0.659 [0.397, 1.096] | 1.724 [0.515, 5.769] |
| One or more physical chronic conditions | 1.394 [0.812, 2.394] | 0.811 [0.525, 1.254] | 0.86 [0.572, 1.293] | 1.026 [0.584, 1.802] | 1.016 [0.65, 1.588] | 0.869 [0.539, 1.401] | 1.093 [0.333, 3.587] |
| One or more mental health conditions | **0.401** [0.223, 0.72]** | 0.783 [0.467, 1.314] | 1.071 [0.658, 1.742] | 0.844 [0.435, 1.635] | 0.775 [0.463, 1.296] | 0.852 [0.488, 1.486] | 4.671 [0.519, 42.044] |
| Number of perceived severe symptoms | **1.153* [1.033, 1.288]** | 1.038 [0.943, 1.143] | 1.038 [0.951, 1.134] | 1.071 [0.95, 1.207] | 1.075 [0.976, 1.184] | 1.079 [0.97, 1.2] | 1.024 [0.767, 1.367] |
| Long COVID-19 onset | 1.168 [0.973, 1.401] | **1.198* [1.029, 1.395]** | 1.032 [0.901, 1.182] | 1.109 [0.951, 1.294] | **1.211* [1.03, 1.423]** | 0.974 [0.824, 1.151] | 0.463 [0.062, 3.484] |
| Long COVID-19 duration | 1.038 [0.978, 1.102] | **1.085** [1.03, 1.142]** | 1.027 [0.982, 1.074] | 1.051 [0.999, 1.106] | **1.073** [1.018, 1.132]** | 0.996 [0.942, 1.053] | 0.761 [0.382, 1.516] |
| N | 308 | 367 | 379 | 371 | 378 | 297 | 193 |
| *Red numbers refer to adverse patient experiences, green to beneficial ones; N varies by barrier as only applicable participants are included in the analysis* * p-value < 0.05, ** p-value < 0.01, *** p-value < 0.001 | | | | | | | |

|  | ***Appropriateness (II/II)*** | | | | |
| --- | --- | --- | --- | --- | --- |
|  | I had difficulties to be allowed to work part-time during reintegration | Friends and family had difficulties adjusting to my long COVID-19 limitations | My professional environment had difficulties adapting to my long COVID-19 limitations | There is no diagnostic test/biomarker via e.g., a blood count that enables a clear, objective long COVID-19 diagnosis | I had difficulties obtaining a private disability pension |
| ***Independent variables*** | *Odds ratio [95% CI]* | *Odds ratio [95% CI]* | *Odds ratio [95% CI]* | *Odds ratio [95% CI]* | *Odds ratio [95% CI]* |
| Female gender | 1.357 [0.483, 3.809] | 0.633 [0.357, 1.12] | 0.995 [0.526, 1.884] | 0.597 [0.284, 1.256] | *Insufficient number of participants  to whom this barrier was applicable* |
| *Aged 18-30 (base case)* |  |  |  |  |  |
| Aged 31-40 | 0.599 [0.148, 2.423] | 1.036 [0.541, 1.986] | 0.931 [0.437, 1.984] | 1.694 [0.795, 3.606] |  |
| Aged 41-50 | 0.374 [0.096, 1.453] | 0.892 [0.472, 1.686] | 0.988 [0.478, 2.041] | 1.818 [0.865, 3.82] |  |
| Aged 51-60 | 0.673 [0.159, 2.843] | 1.303 [0.661, 2.571] | 1.576 [0.704, 3.525] | **2.882* [1.245, 6.671]** |  |
| Older than 60 | *n/a* | 0.902 [0.27, 3.016] | 0.417 [0.047, 3.714] | 0.901 [0.238, 3.405] |  |
| Living in a rural area | 1.571 [0.743, 3.325] | 1.341 [0.87, 2.065] | 1.121 [0.685, 1.836] | 1.278 [0.746, 2.188] |  |
| Medical proximity | **2.672* [1.205, 5.926]** | **0.633* [0.407, 0.984]** | **1.887* [1.099, 3.24]** | 0.663 [0.387, 1.137] |  |
| Complementary health insurance | 1.158 [0.496, 2.703] | 1.411 [0.884, 2.252] | 1.056 [0.617, 1.806] | 0.632 [0.36, 1.111] |  |
| Household financial situation good or very good | 0.798 [0.292, 2.179] | 0.719 [0.412, 1.252] | **0.377** [0.201, 0.707]** | 0.802 [0.413, 1.557] |  |
| *Household financial situation* *medium (base case)* |  |  |  |  |  |
| Household financial situation bad or very bad | 1.885 [0.84, 4.231] | 1.503 [0.942, 2.399] | 1.088 [0.624, 1.899] | 0.903 [0.5, 1.633] |  |
| Living in a state with central coordination | 0.489 [0.223, 1.075] | 0.874 [0.543, 1.408] | 1.019 [0.579, 1.795] | **0.526* [0.296, 0.933]** |  |
| *Education below A-level equivalent (base case)* |  |  |  |  |  |
| Education at A-level equivalent | 1.303 [0.392, 4.329] | 1.449 [0.74, 2.835] | 0.849 [0.377, 1.913] | **2.603* [1.017, 6.659]** |  |
| Education higher than A-level equivalent | 0.585 [0.223, 1.531] | 0.868 [0.506, 1.486] | 1.086 [0.56, 2.105] | 0.914 [0.464, 1.8] |  |
| Migration background | 1.386 [0.434, 4.426] | 1.044 [0.546, 1.998] | 1.003 [0.456, 2.209] | 0.657 [0.305, 1.414] |  |
| Living in a relationship | 0.748 [0.322, 1.738] | 1.043 [0.667, 1.632] | 0.737 [0.435, 1.249] | 0.804 [0.458, 1.413] |  |
| One or more physical chronic conditions | 0.636 [0.29, 1.393] | 1.25 [0.815, 1.918] | 1.062 [0.639, 1.766] | 0.703 [0.415, 1.19] |  |
| One or more mental health conditions | 0.69 [0.281, 1.696] | 0.778 [0.473, 1.277] | 0.6 [0.336, 1.073] | 1.36 [0.722, 2.563] |  |
| Number of perceived severe symptoms | 1.079 [0.906, 1.285] | **1.117* [1.017, 1.226]** | **1.139* [1.018, 1.275]** | 1.046 [0.934, 1.173] |  |
| Long COVID-19 onset | 1.096 [0.903, 1.33] | **1.183* [1.037, 1.35]** | 1.047 [0.916, 1.195] | 1.107 [0.954, 1.284] |  |
| Long COVID-19 duration | 1.012 [0.952, 1.076] | **1.060** [1.015, 1.106]** | 1.031 [0.987, 1.077] | 1.032 [0.983, 1.083] |  |
| N | 141 | 382 | 297 | 373 | 58 |
| *Red numbers refer to adverse patient experiences, green to beneficial ones; N varies by barrier as only applicable participants are included in the analysis* * p-value < 0.05, ** p-value < 0.01, *** p-value < 0.001 | | | | | |

Table A4. Regression results for individual facilitators for adult long COVID-19 patients in Austria

|  | ***Facilitators (I/II)*** | | | | | | |
| --- | --- | --- | --- | --- | --- | --- | --- |
|  | My social environment and family were a great support to me | My professional environment was very understanding | The Long Covid Austria Facebook group provided useful information | The exchange with other patients helped me | My GP coordinated my treatment | GPs encouraged me in the perception of my illness | Specialists encouraged me in the perception of my illness |
| ***Independent variables*** | *Odds ratio [95% CI]* | *Odds ratio [95% CI]* | *Odds ratio [95% CI]* | *Odds ratio [95% CI]* | *Odds ratio [95% CI]* | *Odds ratio [95% CI]* | *Odds ratio [95% CI]* |
| Female gender | 1.185 [0.661, 2.127] | 1.013 [0.584, 1.756] | 0.919 [0.466, 1.813] | 1.094 [0.597, 2.007] | 1.152 [0.575, 2.312] | 1.146 [0.624, 2.102] | 0.644 [0.367, 1.131] |
| *Aged 18-30 (base case)* |  |  |  |  |  |  |  |
| Aged 31-40 | 0.98 [0.466, 2.062] | 1.425 [0.732, 2.774] | 0.728 [0.333, 1.591] | 1.545 [0.782, 3.055] | 0.522 [0.237, 1.15] | 0.699 [0.348, 1.403] | 1.245 [0.645, 2.403] |
| Aged 41-50 | 0.537 [0.267, 1.082] | 1.569 [0.823, 2.991] | 1.134 [0.524, 2.455] | 1.296 [0.668, 2.517] | 0.656 [0.313, 1.376] | 0.749 [0.394, 1.427] | 1.197 [0.638, 2.247] |
| Aged 51-60 | **0.447* [0.213, 0.937]** | 1.591 [0.791, 3.202] | 1.085 [0.47, 2.503] | 1.474 [0.714, 3.04] | 0.604 [0.267, 1.365] | 0.901 [0.445, 1.825] | 1.116 [0.565, 2.205] |
| Older than 60 | 1.477 [0.338, 6.443] | **6.567** [1.783, 24.186]** | 0.789 [0.161, 3.877] | 2.954 [0.748, 11.669] | 3.2 [0.868, 11.794] | 1.522 [0.478, 4.85] | 1.784 [0.507, 6.28] |
| Living in a rural area | 1.154 [0.729, 1.825] | 0.875 [0.568, 1.347] | 0.917 [0.549, 1.533] | 0.822 [0.518, 1.304] | 1.061 [0.608, 1.853] | 1.045 [0.653, 1.674] | 0.791 [0.506, 1.237] |
| Medical proximity | 1.053 [0.655, 1.693] | 0.901 [0.576, 1.409] | 1.012 [0.606, 1.69] | 0.781 [0.489, 1.248] | 1.371 [0.785, 2.394] | 1.072 [0.662, 1.735] | 0.704 [0.451, 1.1] |
| Complementary health insurance | 1.085 [0.656, 1.796] | 1.155 [0.718, 1.857] | 0.945 [0.537, 1.664] | 0.842 [0.509, 1.393] | 1.137 [0.618, 2.091] | 1.075 [0.647, 1.787] | **1.859* [1.149, 3.007]** |
| Household financial situation good or very good | 0.978 [0.518, 1.844] | 0.966 [0.551, 1.694] | 0.573 [0.283, 1.159] | 0.583 [0.318, 1.067] | 1.401 [0.701, 2.797] | **2.031* [1.108, 3.720]** | 1.074 [0.611, 1.888] |
| *Household financial situation* *medium (base case)* |  |  |  |  |  |  |  |
| Household financial situation bad or very bad | **0.448** [0.274, 0.734]** | 0.794 [0.497, 1.269] | 0.852 [0.498, 1.458] | 0.948 [0.577, 1.557] | 1.177 [0.649, 2.137] | 1.507 [0.896, 2.535] | 0.953 [0.602, 1.509] |
| Living in a state with central coordination | 1.137 [0.678, 1.907] | 1.566 [0.963, 2.547] | 0.806 [0.434, 1.495] | 0.828 [0.504, 1.361] | 1.376 [0.753, 2.515] | 1.065 [0.63, 1.8] | 0.668 [0.415, 1.075] |
| *Education below A-level equivalent (base case)* |  |  |  |  |  |  |  |
| Education at A-level equivalent | 0.667 [0.338, 1.318] | 0.974 [0.49, 1.939] | **2.291* [1.029, 5.1]** | **2.663** [1.265, 5.605]** | 1.307 [0.593, 2.88] | 1.581 [0.765, 3.268] | 0.599 [0.301, 1.19] |
| Education higher than A-level equivalent | 1.196 [0.679, 2.106] | 1.089 [0.626, 1.895] | 1.54 [0.834, 2.842] | 1.392 [0.787, 2.463] | 0.646 [0.335, 1.246] | 0.806 [0.446, 1.457] | 0.747 [0.429, 1.300] |
| Migration background | 1.012 [0.497, 2.059] | 0.863 [0.435, 1.713] | 0.962 [0.418, 2.215] | 0.823 [0.423, 1.602] | 1.08 [0.508, 2.297] | 1.173 [0.586, 2.348] | **2.040* [1.053, 3.953]** |
| Living in a relationship | **1.629* [1.024, 2.592]** | 1.312 [0.835, 2.06] | 1.283 [0.755, 2.179] | **1.661* [1.047, 2.633]** | 0.86 [0.494, 1.496] | 1.19 [0.728, 1.945] | **1.605* [1.020, 2.524]** |
| One or more physical chronic conditions | 0.846 [0.537, 1.332] | 0.814 [0.526, 1.258] | 1.019 [0.605, 1.716] | 1.474 [0.922, 2.354] | 0.935 [0.540, 1.620] | 1.102 [0.687, 1.768] | 1.21 [0.784, 1.866] |
| One or more mental health conditions | 0.887 [0.522, 1.507] | 1.205 [0.726, 2.000] | 0.622 [0.346, 1.12] | 0.831 [0.491, 1.408] | 0.630 [0.311, 1.276] | 0.825 [0.473, 1.441] | **1.792* [1.060, 3.029]** |
| Number of perceived severe symptoms | 1.000 [0.907, 1.103] | 0.954 [0.868, 1.048] | 0.988 [0.885, 1.102] | 1.044 [0.946, 1.154] | 0.944 [0.844, 1.057] | 1.001 [0.905, 1.107] | 0.968 [0.882, 1.063] |
| Long COVID-19 onset | 1.082 [0.952, 1.229] | 1.074 [0.939, 1.228] | 0.973 [0.772, 1.226] | 1.055 [0.908, 1.226] | 0.929 [0.774, 1.115] | **0.841* [0.730, 0.968]** | 0.963 [0.831, 1.117] |
| Long COVID-19 duration | 1.019 [0.978, 1.062] | 1.001 [0.958, 1.046] | 0.999 [0.925, 1.078] | 1.039 [0.988, 1.091] | 0.972 [0.915, 1.031] | **0.929** [0.887, 0.974]** | 0.975 [0.93, 1.023] |
| N | 383 | 347 | 283 | 357 | 280 | 314 | 355 |
| *Red numbers refer to adverse patient experiences, green to beneficial ones; N varies by facilitator as only applicable participants are included in the analysis* * p-value < 0.05, ** p-value < 0.01, *** p-value < 0.001 | | | | | | | |

|  | ***Facilitators (II/II)*** | | |
| --- | --- | --- | --- |
|  | Doctors offered to shorten waiting times by switching to a private practice | Doctors offered to shorten waiting times through private co-payments | Telemedicine facilitated my treatment |
| ***Independent variables*** | *Odds ratio [95% CI]* | *Odds ratio [95% CI]* | *Odds ratio [95% CI]* |
| Female gender | 0.509 [0.167, 1.549] | 1.042 [0.182, 5.962] | 1.642 [0.709, 3.801] |
| *Aged 18-30 (base case)* |  |  |  |
| Aged 31-40 | 0.628 [0.173, 2.283] | 3.319 [0.428, 25.714] | 0.55 [0.21, 1.443] |
| Aged 41-50 | 0.475 [0.136, 1.665] | 2.154 [0.235, 19.727] | 0.521 [0.211, 1.286] |
| Aged 51-60 | **0.104** [0.02, 0.541]** | 0.610 [0.068, 5.458] | **0.322* [0.119, 0.869]** |
| Older than 60 | 0.89 [0.084, 9.446] | 3.064 [0.055, 169.579] | 0.212 [0.035, 1.274] |
| Living in a rural area | 1.238 [0.492, 3.113] | 1.351 [0.340, 5.370] | 1.405 [0.743, 2.654] |
| Medical proximity | 0.819 [0.29, 2.318] | 1.459 [0.305, 6.981] | 0.677 [0.342, 1.339] |
| Complementary health insurance | 1.622 [0.62, 4.246] | 2.714 [0.635, 11.612] | 1.391 [0.694, 2.787] |
| Household financial situation good or very good | 1.415 [0.417, 4.793] | 6.574 [0.654, 66.08] | 0.652 [0.272, 1.565] |
| *Household financial situation* *medium (base case)* |  |  |  |
| Household financial situation bad or very bad | 1.858 [0.691, 4.993] | 0.678 [0.142, 3.243] | 0.830 [0.422, 1.631] |
| Living in a state with central coordination | 0.955 [0.367, 2.484] | 1.236 [0.24, 6.363] | **0.219*** [0.101, 0.474]** |
| *Education below A-level equivalent (base case)* |  |  |  |
| Education at A-level equivalent | 1.286 [0.329, 5.018] | 4.908 [0.402, 59.947] | 1.523 [0.55, 4.212] |
| Education higher than A-level equivalent | 2.76 [0.815, 9.343] | **31.241** [2.345, 416.12]** | 1.573 [0.653, 3.792] |
| Migration background | 3.393 [0.858, 13.418] | 2.279 [0.32, 16.244] | 1.655 [0.689, 3.974] |
| Living in a relationship | 1.459 [0.507, 4.197] | 1.812 [0.344, 9.549] | 0.585 [0.304, 1.126] |
| One or more physical chronic conditions | **3.704* [1.276, 10.753]** | 3.073 [0.744, 12.692] | 1.657 [0.877, 3.131] |
| One or more mental health conditions | 1.451 [0.432, 4.874] | 2.363 [0.28, 19.908] | 1.092 [0.518, 2.302] |
| Number of perceived severe symptoms | 1.056 [0.857, 1.300] | **1.680* [1.126, 2.507]** | 0.895 [0.777, 1.032] |
| Long COVID-19 onset | 0.931 [0.726, 1.193] | 0.908 [0.577, 1.429] | 1.056 [0.844, 1.323] |
| Long COVID-19 duration | 0.973 [0.900, 1.051] | 0.900 [0.773, 1.048] | 1.058 [0.982, 1.14] |
| N | 103 | 72 | 184 |
| *Red numbers refer to adverse patient experiences, green to beneficial ones; N varies by facilitator as only applicable participants are included in the analysis* * p-value < 0.05, ** p-value < 0.01, *** p-value < 0.001 | | | |

Table A5. Regression results for individual unmet healthcare needs for adult long COVID-19 patients in Austria

|  | ***Unmet healthcare needs*** | | |
| --- | --- | --- | --- |
|  | **GP** | **Specialist** | **Hospital** |
| ***Independent variables*** | *Odds ratio [95% CI]* | *Odds ratio [95% CI]* | *Odds ratio [95% CI]* |
| Female gender | 0.761 [0.411, 1.408] | 0.733 [0.367, 1.462] | 0.765 [0.404, 1.446] |
| *Aged 18-30 (base case)* |  |  |  |
| Aged 31-40 | 0.571 [0.282, 1.158] | 0.504 [0.222, 1.143] | 0.527 [0.253, 1.097] |
| Aged 41-50 | 0.808 [0.406, 1.609] | 0.723 [0.323, 1.614] | 0.684 [0.334, 1.398] |
| Aged 51-60 | 0.751 [0.358, 1.575] | 0.53 [0.225, 1.249] | 0.651 [0.302, 1.403] |
| Older than 60 | **0.196* [0.043, 0.892]** | 0.257 [0.06, 1.104] | 0.743 [0.184, 3.006] |
| Living in a rural area | **1.708* [1.063, 2.747]** | **1.788* [1.038, 3.078]** | 1.46 [0.895, 2.381] |
| Medical proximity | 0.938 [0.581, 1.516] | 1.331 [0.768, 2.307] | **1.677* [1.014, 2.771]** |
| Complementary health insurance | 0.634 [0.382, 1.053] | 0.782 [0.446, 1.368] | 0.837 [0.496, 1.414] |
| Household financial situation good or very good | 0.611 [0.337, 1.107] | **0.483* [0.26, 0.898]** | **0.518* [0.284, 0.947]** |
| *Household financial situation* *medium (base case)* |  |  |  |
| Household financial situation bad or very bad | 1.264 [0.764, 2.09] | 1.615 [0.894, 2.918] | 1.193 [0.708, 2.01] |
| Living in a state with central coordination | 0.728 [0.438, 1.211] | 0.652 [0.373, 1.14] | **0.476** [0.283, 0.8]** |
| *Education below A-level equivalent (base case)* |  |  |  |
| Education at A-level equivalent | 0.914 [0.446, 1.873] | 1.475 [0.656, 3.314] | 1.029 [0.493, 2.144] |
| Education higher than A-level equivalent | 1.723 [0.949, 3.13] | **2.061* [1.048, 4.053]** | 1.548 [0.837, 2.864] |
| Migration background | 1.046 [0.516, 2.12] | 1.437 [0.601, 3.434] | 0.859 [0.419, 1.763] |
| Living in a relationship | 0.879 [0.544, 1.422] | 0.906 [0.526, 1.561] | 0.931 [0.567, 1.529] |
| One or more physical chronic conditions | 1.079 [0.674, 1.728] | 1.459 [0.846, 2.514] | 1.115 [0.686, 1.813] |
| One or more mental health conditions | 0.964 [0.562, 1.653] | 0.613 [0.338, 1.112] | 0.889 [0.511, 1.547] |
| Number of perceived severe symptoms | **1.177** [1.062, 1.305]** | **1.197** [1.066, 1.345]** | **1.225*** [1.101, 1.363]** |
| Long COVID-19 onset | 1.081 [0.924, 1.265] | 1.148 [0.985, 1.338] | 1.17 [0.992, 1.379] |
| Long COVID-19 duration | 1.043 [0.99, 1.099] | **1.064* [1.012, 1.12]** | **1.059* [1.002, 1.118]** |
| N | 387 | 387 | 387 |
| *Red numbers refer to adverse patient experiences, green to beneficial ones* * p-value < 0.05, ** p-value < 0.01, *** p-value < 0.001 | | |  |
